# In vitro and preclinical evaluation of the antifungal activity of 6-methoxy-1 H-indole-2-carboxylic acid produced by *Bacillus toyonensis* strain OQ071612 formulated as nanosponge hydrogel

**DOI:** 10.1186/s12934-025-02688-y

**Published:** 2025-04-01

**Authors:** Sayed E. El-Sayed, Neveen A. Abdelaziz, Ghadir S. El-Housseiny, Khaled M. Aboshanab

**Affiliations:** 1 Department of Microbiology and Immunology, Faculty of Pharmacy, Sixth of October City, Ahram Canadian University, 6 October city, Giza 12451 Egypt; 2 Department of Microbiology and Immunology, Faculty of Pharmacy, Ain Shams University, Cairo, 11566 Egypt

**Keywords:** Antifungal, Nanosponges, Hydrogel, *Bacillus toyonensis*, Box Behnken design

## Abstract

**Background:**

In a previous study, 6-methoxy-1 H-indole-2-carboxylic acid (MICA) was isolated from the culture broth of *Bacillus toyonensis* strain OQ071612 soil isolate in our laboratory, and it demonstrated promising antifungal activities. The current study was designed to create a nanosponge (NS)-hydrogel (HG)-containing MICA followed by in vitro and preclinical evaluation for potential clinical use in the topical treatment of mycotic infections.

**Results:**

The enhanced NS formula was created using the Box Behnken Design (BBD), with independent process parameters including polyvinyl alcohol percentage (w/v%), homogenization time, speed and polymer: linker ratio. Dependent parameters were particle size (PS), polydispersity index (PDI), and entrapment efficiency percent (EE%). A hydrogel was formulated from the NS. In vitro drug release data indicated that the hydrogel best matched Higuchi’s kinetic release model. The formulated NS-HG was stable and when compared to fluconazole, it exhibited increased antimycotic activity against *C. albicans*. An in vivo investigation revealed that MICA-NS-HG enhanced survival rates, wound gap repair, wound reduction, and inflammation inhibition. Masson’s trichrome staining and histological analyses revealed increased collagen deposition and improved healing. Moreover, MICA hydrogel exhibited 1.5-fold greater permeability through rat skin compared to the control, 1% isoconazole.

**Conclusion:**

The NS-HG formulation is a viable vehicle for better and more effective topical release of MICA. These findings represent a significant advancement in the formulation of MICA derived from naturally occurring soil bacteria.

**Supplementary Information:**

The online version contains supplementary material available at 10.1186/s12934-025-02688-y.

## Background

The prevalence and severity of fungal infections have surged in the past few years due to the increased use of immunosuppressive therapies and the emergence of epidemics. A major concern in the treatment of fungal infections is the growing resistance of fungal strains to antifungal medications. This resistance complicates the management of infections and limits therapeutic options, leading to prolonged illness and higher mortality rates [1]. Currently, there are three main classes of registered systemic antifungals– azoles, echinocandins, and polyenes [2]. Among these three groups of antifungal agents, limitations exist with regards to their use which include spectrum of activity, resistance, toxicity, suboptimal pharmacokinetics, drug–drug interactions, and poor bioavailability [3]. In addition, available topical antifungal agents are categorized into polyenes, azoles, and echinocandins. However, their therapeutic efficacy is often compromised due to their high lipophilicity and low aqueous solubility, which hinder their effective delivery. These physicochemical properties contribute to insufficient drug deposition at target skin sites, limited penetration across the stratum corneum, and consequently, reduced bioavailability. To overcome these limitations, the development of nanostructured drug carriers has emerged as a promising strategy to enhance drug retention, skin penetration, and overall therapeutic efficacy of topical antifungal treatments [4].

The emergence of COVID-19 has further complicated the management of fungal infections, particularly for immunodeficient patients [5]. Studies proved a higher frequency of *C. albicans* and *Aspergillus fumigatus* coinfections in critically ill COVID-19 patients [5, 6]. Dermatologists prefer topical formulations for the management of superficial mycotic infections over parenteral or peroral formulations due to their improved patient compliance, thanks to their non-invasive design, effective targeting qualities, low adverse effects, and ease of administration [7]. Hydrogels are becoming more and more common among topical dosage forms because of their capacity to swell, their ability to control drug release together, and their strength of adhesion [8]. Polysaccharide biopolymers, known for their safety, non-carcinogenic, and non-allergenic properties, play a crucial role in medicine. Hence, the popularity of biodegradable, safe β-Cyclodextrins (β-CD) has grown due to their ability to interact with drugs non-covalently [9].

A novel formulation called nanosponges (NS) is used to encapsulate nanoparticles. It has a porous, non-collapsible, sponge-like structure [10]. As it has the advantages of microsponges and nanoscale vesicular structures, it is mostly utilized in many pharmaceutical industry purposes [11]. The permeable structure not only enables us to capture a broad spectrum of active chemicals but also influences the release pattern [11]. Excellent characteristics of cyclodextrin NSs include their easy preparation, their capacity to form inclusion complexes, and their ability to improve drug aqueous solubility [12]. When combined with hydrogels, cyclodextrin NSs offer exceptional advantages due to their three-dimensional porous structure. These benefits include enhanced skin retention, improved patient compliance, reduced dosage requirements, and fewer side effects [13]. Response surface methodology (RSM) is a powerful optimization technique that uses mathematical models to optimize processes influenced by multiple components [14]. By employing lower-order polynomial equations, RSM designs experiments to save time and materials by reducing the total number of possible combinations [15].

*Bacillus toyonensis* is a member of the *Bacillus cereus* group—it is a spore-forming, Gram-positive organism [16]. It has enormous economic significance; for example, *Bacillus toyonensis* BCT-7112 spores have been utilized as probiotic supplements in animal nutrition [17]. It was also previously reported to exhibit plant growth promotion, biodegradation, and probiotic properties [18]. Its biocontrol properties are rarely reported; however, Wang et al. reported its toyoncin-producing ability. Toyoncin, commonly produced by bacteria, e.g., *Bacillus spp and Lactobacillus spp*, is an indole carboxylic acid derivative that displays antibacterial action against two major foodborne pathogens, *B. cereus* (through limiting the growth of its spores) *and Listeria monocytogenes* [19]. It also causes cell membrane damage [19]. Indole carboxylic acid derivatives have been previously reported for their antifungal activity [20] and have previously been documented to be produced by algae and bacteria, such as *Micromonospora* sp. and *Sandaracinus amylolyticus* [21–23].

In our earlier study, a novel antimycotic metabolite 6-methoxy-1 H-indole-2-carboxylic acid (MICA) from *B. toyonensis* isolate OQ071612 was isolated, optimized, purified and its antifungal activity was evaluated against *C. albicans* ATCC 10,231 and *Aspergillus niger* clinical isolates [24] However, it was not either formulated in appropriate pharmaceutical formulation to enable its medical application or clinically evaluated. Taking the several advantages of the novel NS formulations particularly for topical application as previously reported [10, 11, 24], this study aimed to formulate a (NS)-hydrogel (HG)-containing MICA followed by in vitro and preclinical evaluation of its antifungal activity for potential clinical use in the topical treatment of mycotic infections in humans.

## Materials and methods

### Chemicals and culture media

Middle East Company (Cairo, Egypt) supplied β-cyclodextrin (β-CD), diphenyl carbonate (DPC), carbopol 940, and poloxamer 188. El-Nasr Pharmaceuticals (ADWIC, Cairo, Egypt) provided the carboxymethyl cellulose (CMC), sodium alginate, hydroxyl propyl methyl cellulose (HPMC E4), and tween 80. Fluconazole was sourced from Sedico Pharmaceutical Company (Giza, Egypt), while Isoconazole (ISN) 1% was from Al-Esraa Pharmaceutical Optima Co. (Cairo, Egypt). Sabouraud dextrose agar (SDA) was obtained from Oxoid Ltd (Basingstoke, England), and collagenase 0.6 IU from Abbott Co. (Wiesbaden, Germany).

### Recovery of MICA

As previously reported, MICA was extracted from the culture broth of *B. toyonensis* [24]. This bacterial isolate was put in the Culture Collection Ain Shams University (CCASU) under the code CCASU-OQ071612 (http://ccinfo.wdcm.org/collection/by_id/1186). Additionally, it has an NCBI GenBank accession number OQ071612.

### Formulation of MICA-loaded nanosponges (NS)

The NS were prepared by different ratios of diphenyl carbonate and β-cyclodextrin, utilizing the emulsion solvent diffusion technique [25]. This procedure included generating two distinct phases: a dispersed phase and a continuous phase. Utilizing a homogenizer (Ultra Turrax, Germany), the dispersed phase was created by ultrasonically swirling various ratios of β-cyclodextrin and crosslinking diphenyl carbonate with 105 µg MICA in 20 mL dichloromethane for 10 min. Next, in a water bath heated to 60 °C, 150 mL of distilled water was mixed with 0.5% w/v polyvinyl alcohol (PVA) to create a continuous aqueous phase.

Using a syringe, we gradually added the dispersed phase to the continuous phase while simultaneously combining the mixture at 35 °C for 10 min using probe-sonication (Fisher Scientific, Waltham, MA, USA). Subsequently, the mixture was allowed to homogenize. After separating the solid mass from the dispersion (the NSs created), the unreacted β-CD residues were removed with a 50:50 ethanol/methanol wash and 0.45 μm filter paper [26]. After further filtering, it was put into glass vials and pre-frozen at -80 °C for 12 h. Mannitol was used as a cryoprotectant, and it was lyophilized for two days at -48 °C and a pressure of 0.37 mbar. The final powder was sealed in airtight receptacles for storage [27].

The optimization procedure made use of the Box-Behnken design. According to previous studies, four factors which proved to have a significant effect on our responses were included in our model [28, 29]. The ratio of the crosslinker, diphenyl carbonate, and the polymer, β-cyclodextrin, as well as the homogenization time, speed, and PVA percentage (w/v %) were independent factors. Three values, PDI, PS (nm), and entrapment efficiency % (EE), were the responses to be measured. The levels were as follows: Factor A: β-CD: diphenyl carbonate molar ratio: 1:2, 1:4, 1:10; Factor B: Homogenization speed (rpm): 10,000, 12,500, 15,000; Factor C: Homogenization time (min):10, 12, 14; Factor D: Polyvinyl alcohol (%w/v):0.3, 0.4, 0.5. Our Target was to minimize Particle size and Polydispersity index but maximize Entrapment efficiency %. The Box-Behnken design, as displayed in Table 1, called for 29 experimental runs that produced 3 quadratic models [14].

**Table 1:** Runs created by the Box-Behnken design and the observed responses

Run	A: Polymer-linker ratio	B: Homogenization speed	C: Homogenization time	D: PVA%	Particle size	PDI	EE
1	1:10	12,500	14	0.4	408	0.4	88
2	1:4	12,500	12	0.4	375	0.26	87
3	1:4	12,500	10	0.5	384	0.24	88
4	1:4	10,000	10	0.4	387	0.36	91
5	1:4	15,000	12	0.3	400	0.4	85.4
6	1:4	10,000	12	0.5	392	0.31	89.2
7	1:10	12,500	10	0.4	405	0.33	84
8	1:2	12,500	12	0.3	424	0.51	88
9	1:4	12,500	14	0.3	378	0.44	88.5
10	1:4	12,500	12	0.4	375	0.21	87.8
11	1:4	12,500	12	0.4	375	0.21	87.8
12	1:4	12,500	12	0.4	375	0.21	87.8
13	1:10	15,000	12	0.4	418	0.53	84.4
14	1:10	10,000	12	0.4	415	0.56	84.9
15	1:4	12,500	12	0.4	375	0.23	87.78
16	1:2	12,500	10	0.4	422	0.42	88.6
17	1:4	10,000	14	0.4	385	0.3	88.9
18	1:2	15,000	12	0.4	430	0.64	82
19	1:4	15,000	10	0.4	398	0.25	85
20	1:4	15,000	14	0.4	394	0.45	87
21	1:2	12,500	12	0.5	426	0.57	84
22	1:4	12,500	14	0.5	379	0.22	88.8
23	1:2	10,000	12	0.4	429	0.6	89
24	1:4	10,000	12	0.3	390	0.5	89.2
25	1:4	15,000	12	0.5	402	0.45	85.02
26	1:2	12,500	14	0.4	420	0.5	84.56
27	1:10	12,500	12	0.3	411	0.59	84.1
28	1:4	12,500	10	0.3	382	0.26	88.45
29	1:10	12,500	12	0.5	412	0.3	86.55

### Physicochemical properties of the formulated MICA-NS

Using the Malvern-Nano ZS-Zeta sizer (Malvern Instruments Ltd., Worcestershire, UK) and dynamic light scattering (DLS) approach, the average PS, PDI, and ZP of the prepared MICA-loaded NSs were assessed. All samples were suitably diluted with distilled water before being subjected to three minutes of ultrasonography processing to separate adherents. The samples were measured in triplicate. The entrapment efficiency (% EE**)** was calculated as formerly reported [25] using Eq. 1. The MICA concentration was calculated from the prepared standard curve. Every measurement was taken thrice, and the averages were noted. The drug loading capacity % (DL%) was calculated using Eq. 2 [30]:

1 $${\rm{\% EE}} = {{{\rm{Actual drug content in NS}}} \over \matrix{\,\,\,\,\,\,{\rm{Theoretical content}} \hfill \cr {\rm{(content of MICA initially added)}} \hfill \cr} } \times 100$$

undefined

2 $$\% DL = {{Weight\>of\>MICA\>in\>NS\>x\>100} \over {\>weight\>of\>NS}}$$

of\>MICA\>in\>NS\>x\>100} \over {\>weight\>of\>NS}}]]>

### Analysis using scanning electron microscopy (SEM)

Utilizing a Joel JSM-SEM (model: JSM6330 LV, Tokyo, Japan), the surface morphology of the optimal NS formula was visualized. The gold-coated optimized MICA-NS was positioned atop the SEM stubs. To ascertain the spherical 3D structure of the NS produced, areas were scanned, recorded, and processed at different magnifications [31].

### Analysis using fourier transform infra-red (FTIR) spectroscopy

FTIR analysis was performed for MICA-NS and pure MICA to verify the absence of interactions between MICA and the polymer. The material was properly diluted with crystalline KBr (1:10 w/w) and then pressed into a translucent film. The film was mounted on a sample holder, and spectrum management software was utilized to record spectra across various frequencies between 4000 and 400 cm-1 [31, 32].

### Differential scanning calorimetry

Differential scanning calorimetry (DSC) using TA Instruments, California (Discovery DSC25 series) was undertaken. The instrument was equipped with a Refrigerated Cooling System 90 (RCS 90, TA Instruments, New Castle, USA) and TRIOS software. For the enthalpy heat and melting point, indium was used for graduation of the DSC. Under a nitrogen purge (350 mL/min), a heating rate of 10 °C/min was retained between 30 °C and 400 °C and an empty pan was used as a reference [30].

### Creation of a topical hydrogel with the MICA-NS

Several gel formers at different proportions (0.2%, 0.5%, 0.8%, 1%, 1.2%, and 1.5% w/v) to incorporate the optimized MICA-loaded NS into a hydrogel formulation were investigated. Our objective was to identify the most effective gel former as previously described [33]. A magnetic stirrer set at 600 rpm was used to disperse the gelling agent uniformly after a predetermined amount was added and dissolved in a mixture of 100 mL of 30:70 propylene glycol: distilled water. A 15-min stagnation period was then allowed to liberate any trapped air. Two milliliters of triethanolamine were added to attain clarity and a pH of 6.7–6.9 [34]. Subsequently, distilled water was added to the gel to reach a total volume of 100 ml after adding one gram of methylparaben for preservation purposes. The dispersion was continuously stirred at 600 rpm for approximately six hours until a smooth gel without any lumps was formed. It was then left overnight to achieve complete hydration and then kept for later experiments at 5 °C in tightly sealed cases [33].

### NS-loaded hydrogel physical parameters

For uniformity, each NS-loaded hydrogel was visually examined and spreadability was assessed. A glass plate (5 × 5 cm) with 100 mg of the produced hydrogel was employed for the gel loading. Another glass plate of equal size was placed on top at a height of five centimeters. Applying a steady weight of 0.5 kg to the upper plate for 1 min, and the spread circle’s diameter (cm) after removing the weight was observed. This test confirmed that the hydrogel could flow out of collapsible tubes under the application of a consistent 500 g weight for 10 s [33]. Extrudability was measured in g/s for each formula, and the analysis was done in triplicate. A perforated aluminum foil, previously weighed, with 0.5 g of hydrogel to assess its swelling behavior was filled. During the experiment, the weight increased over time as the hydrogel soaked in 10 mL of phosphate buffer was measured. To eliminate excess swollen media, the aluminum foil was allowed to air for approximately 15 min before measurement. The measurements were stopped when three consecutive readings remained constant. Samples were retrieved from the beakers at 30, 60, and 120 min. They were then left on a dry surface for a while before being weighed again and the results were computed using Eq. (3) [33]:

3 $$\:\:\:\:\:\:\:\:\:\:\:\:\:\:\:\text{S}\text{w}\text{e}\text{l}\text{l}\text{i}\text{n}\text{g}\:\text{i}\text{n}\text{d}\text{e}\text{x}\:\left(\text{S}\text{W}\right)\text{\%}=\frac{\text{W}\text{t}\:-\text{W}\text{o}}{\text{W}\text{o}}\:\text{X}\:100\:\:\:\:\:\:\:\:\:\:\:$$

(SW) %: swelling index, W_t_: weight of gel at time t and W_o_: weight of gel at zero time.

At 25 °C, the pH of the chosen hydrogel was measured, and a rheometer (AMETEK Brookfield, PVS-223HC-HT, East Lyme, USA) was utilized to examine the viscosity of the optimized formula [30].

### Determination of drug content

About fifty milligrams of the selected hydrogel in 100 ml of phosphate buffer (pH 7.4) were dissolved and agitated for two hours. Following this, the mixture was subjected to two minutes of bath sonication. The goal of this approach was to maximize medication solubility when subjected to mechanical shaking. After filtration, we measured the solution’s absorbance at 273 nm. Using the standard curve of MICA, the amount of drug present in the hydrogel was determined.

### Evaluation of the in vitro release

Using the paddle approach and the USP dissolving equipment II, MICA was released from MICA-NS-HG. The paddles rotated at 50 rpm and the temperature was kept at 37 ± 0.5 °C to simulate the condition of human skin [33, 35, 36]. Ten mg of the optimized MICA-NS-hydrogel were loaded into dialysis bags which were connected with paddles and placed in phosphate buffer (pH 7.4). Following time intervals of 0, 1, 3, 5, 7, 9, 11, 13, 15, and 24 h, 3 mL were sampled and replaced with an equivalent amount of buffer. Following filtration, the drug concentration at λ 273 nm was measured using spectrophotometry. By suiting the data into multiple kinetic models, the mechanism of drug release and kinetics from the porous NS matrix were determined. As previously reported, the R^2^ value was used to evaluate the drug release mechanism from NSs [37].

### Photodegradation analysis

Photodegradation tests were conducted using a UVA lamp (254–365 nm). First, we evenly packed 40 mg of the hydrogel formulation around the bottom of a beaker. The mixture was then exposed to radiation for 4 h. After the exposure period, we removed the beaker and quantitatively transferred its contents into a calibrated flask (20 ml). Next, we subjected the solution to sonication (15 min) and filtered it using a 0.45 mm membrane filter. Finally, we adjusted the volume to 20 ml and measured it using a UV spectrophotometer. Photodegradation was evaluated following previously established methods [30].

### Stability study

After three months of storage in amber-glass bottles at both room temperature and 5 ± 3 °C, the selected medicated NS-HG formula was examined for physical appearance, viscosity, pH, and drug content [31].

### Evaluation of the antifungal activity in vitro

The wells in Sabouraud dextrose agar (SDA) plates were filled with 100 µL of MICA-NS HG after inoculating the plate with 100 µL of *C. albicans* ATCC 10,231 (adjusted to 0.5 McFarland standard). The experiment also included a placebo, fluconazole at 105 µg/mL, and dimethyl sulfoxide (DMSO) as positive and negative controls, respectively. The inhibition zones in three separate petri dishes were measured as previously reported [38].

### Evaluation of the cytotoxic activity

This was evaluated using MTT assay on the Vero cells cell line (Cercopithecus aethiops) as formerly described [15, 39].

### In vivo evaluation

A total of 40 mature male Wistar albino rats (200–220 g) were used for this experiment. They were bought from Ahram Canadian University’s animal house facility. The rats were adapted for 15 days before the study began. We adhered to the ARRIVE guidelines (https://arriveguidelines.org) and the Care and Use of Laboratory Animals recommendations were followed when handling and housing the animals (accessed on March 20, 2024). The research ethics committee of the Faculty of Pharmacy at Ain Shams University in Cairo examined and approved the entire study (Protocol approval number: ACUC-FP-ASU-RHDIRB2020110301-REC#39).

### Thermal injury model and evaluation of the animal survival rate

Rats were given a third-degree burn wound infection with *C. albicans* ATCC10231, which contained roughly 7.5 × 10^7^ CFU [40]. The rats were split into 8 groups (Groups A-H; five rats each) as described in our previous study [31]. Groups B, C, D, F, G, and H were the same control groups used in our previous study to evaluate ODHP-NS-HG [31]. The studied formulation (1 g of MICA-NS-HG) was applied twice daily for 14 days, starting two hours after the burnt skin became infected. The survival rate of the animals was assessed three days after infection. Mortality rates were calculated after deceased animals were eliminated [41]. Following intraperitoneal anesthesia with a combination of 60 mg kg-1 ketamine and 10 mg kg-1 xylazine, the animals were put to sleep by cervical dislocation. The dorsal skin near the lesion was cautiously detached and preserved in 10% formalin for histological analysis [42].

### Measurement of wound size

The wound’s progress was captured on camera, and Eq. (4) [42] was used to compute the wound contraction percentage based on the wound’s diameter at various periods after the damage:

4 $$\:\text{W}\text{o}\text{u}\text{n}\text{d}\:\text{c}\text{o}\text{n}\text{t}\text{r}\text{a}\text{c}\text{t}\text{i}\text{o}\text{n}\:\text{\%}=\frac{\text{W}\text{t}\:-\text{W}\text{o}}{\text{W}\text{o}}\:\text{X}\:100\:$$

W_t_ (wound area at a given time interval); W_o_ (initial wound area at the onset of the experiment) [43].

### Skin irritation analyses

Any signs of skin irritation for group A rats were documented according to a visual scoring scale as formerly explained [31].

### Histopathological assessment

Histopathological examination was carried out using the standard procedures for sample fixation and staining. To quantify collagen fibers present in the tissues and conduct an objective histological examination, Masson’s trichrome staining method was used. Randomly, six unique, non-overlapping sections from each sample’s dermal layers were selected and analyzed for the relative space occupied by collagen fibers [44].

### Ex vivo skin permeation analysis

Normal abdominal skin of albino rats was used for the skin permeation examination, and Franz diffusion cells with a diffusion area of 3.104 cm and a receptor volume of 22.5 mL were used for the evaluation [45]. Both the formulation (100 mg) and the positive control, ISN1%, were administered equally to the skin. To simulate skin temperature, phosphate buffer (pH 7.4) and 2% v/v DMSO in the lower receptor chamber using a magnetic bead (50 rpm) were continuously mixed at a temperature of 37 ± 0.5 °C. At predefined intervals, we replaced the samples with the same buffer media. After filtration, spectrophotometric analysis at 273 nm for MICA hydrogel and 279.8 nm for ISN1% was performed to determine their contents. To evaluate drug penetration into rat skin, any remaining formula that was visible on the skin’s surface was cleaned with PBS (pH 7.4) [45].

### Evaluation of inflammatory mediators, cytokines and angiogenesis

Blood sampling occurred three days after the study initiation, just before euthanizing the rats. Blood samples from the retro-orbital plexus of each animal in all experimental groups were obtained. During this procedure, the animals were under local anesthesia using lidocaine (4%) [46]. ELISA kit(s) from Elabscience^®^ were used to measure proinflammatory cytokines and inflammatory markers. In the experiment, we combined the relevant antibodies with the collected samples and added them to pre-coated micro-ELISA plates containing rat COX-2, TNF-alpha, VEGF, NF-kB-p105, IL-6, and IL-1 antibodies. The measurement of these respective markers followed previously established protocols [47].

### Statistical analysis

A one-way ANOVA test and Tukey’s multiple comparison test were used to estimate the *p*-values and standard deviation. Design Expert^®^ v. 11.0 was used for records assessment, response surface creation, and model diagnostics.

## Results

### Optimization of MICA-Loaded NS

Table 1 shows the values of the parameters that were studied, and the observed responses. The following are the quadratic BBD model equations that were produced by the software:

Particle size = 862.60698 -504.81982 x polymer-linker ratio − 0.052263 x Homogenization speed − 0.58333 x Homogenization time − 466.26126 x PVA% +898.31081 x polymer-linker ratio^2^ + 2.14919E-006 x Homogenization speed^2^ + 593.24324 x PVA%^2^.

Polydispersity index = 7.54335–4.71059 x polymer-linker ratio − 7.97378E-004 x Homogenization speed − 0.043750 x Homogenization time − 7.18840 x PVA% + 4.37500 x polymer-linker ratio x PVA% + 1.30000E-005 x Homogenization speed x Homogenization time + 2.40000E-004 x Homogenization speed x PVA% -0.25000 x Homogenization time x PVA% + 5.30236 x polymer-linker ratio^2^ + 2.19351E-008 x Homogenization speed^2^ + 6.70946x PVA%^2^.

Entrapment efficiency = 98.33832 + 166.27663 x polymer-linker ratio − 3.60009E-004 x Homogenization speed − 5.51143 x Homogenization time + 22.45417 x PVA% − 3.25000E-003x polymer-linker ratio xHomogenization speed − 5.02500 x polymer-linker ratio x Homogenization time − 80.62500x polymer-linker ratio x PVA% + 2.05000E-004 x Homogenization speed x Homogenization time − 52.24578 x polymer-linker ratio2–7.61730E-008 x Homogenization speed2 + 0.18692x Homogenization time2.

The ANOVA findings for the various responses for the MICA-NS formulation are displayed in Table 2. The models’ significance is demonstrated by the F-values of 238.17, 93.36, and 160.05 (*p*-value < 0.0001) for PS, PDI, and EE%, respectively. Significant model terms include A, B, A², B², D^2^ for PS; A, C, D, AD, BC, BD, CD, A², B², D² for PDI; and A, B, D, AB, AC, AD, BC, A^2^, B², C^2^ for EE%, all of which had *p*-values < 0.05.

**Table 2:** ANOVA analysis and statistical indicators of the Box-Behnken design

MODEL	Sum of Squares	df	Mean Square	F-value	*p*-value
**Particle size**	9848.09	7	1406.87	238.17	< 0.0001
A-polymer-linker ratio	560.33	1	560.33	94.86	< 0.0001
B-Homogenization speed	161.33	1	161.33	27.31	< 0.0001
C-Homogenization time	16.33	1	16.33	2.77	0.1112
D-PVA%	8.33	1	8.33	1.41	0.2482
A^2	8685.85	1	8685.85	1470.46	< 0.0001
B^2	1213.80	1	1213.80	205.49	< 0.0001
D^2	236.76	1	236.76	40.08	< 0.0001
Residual	124.05	21	5.91		
Lack of Fit	124.05	17	7.30		
Pure Error	0.000	4	0.000		
Cor Total	9972.14	28			
**Polydispersity index**	0.52	11	0.048	93.36	< 0.0001
A-polymer-linker ratio	0.023	1	0.023	45.98	< 0.0001
B-Homogenization speed	6.750E-004	1	6.750E-004	1.33	0.2655
C-Homogenization time	0.017	1	0.017	33.15	< 0.0001
D-PVA%	0.031	1	0.031	60.91	< 0.0001
AD	0.031	1	0.031	60.15	< 0.0001
BC	0.017	1	0.017	33.19	< 0.0001
BD	0.014	1	0.014	28.28	< 0.0001
CD	0.010	1	0.010	19.64	0.0004
A^2	0.30	1	0.30	594.40	< 0.0001
B^2	0.13	1	0.13	248.35	< 0.0001
D^2	0.030	1	0.030	59.48	< 0.0001
Residual	8.655E-003	17	5.091E-004		
Lack of Fit	6.735E-003	13	5.181E-004	1.08	0.5217
Pure Error	1.920E-003	4	4.800E-004		
Cor Total	0.53	28			
**Entrapment efficiency**	126.79	11	11.53	160.05	< 0.0001
A-polymer-linker ratio	1.48	1	1.48	20.51	0.0003
B-Homogenization speed	45.55	1	45.55	632.50	< 0.0001
C-Homogenization time	0.042	1	0.042	0.58	0.4555
D-PVA%	0.36	1	0.36	5.01	0.0389
AB	10.56	1	10.56	146.66	< 0.0001
AC	16.16	1	16.16	224.39	< 0.0001
AD	10.40	1	10.40	144.41	< 0.0001
BC	4.20	1	4.20	58.35	< 0.0001
A^2	29.38	1	29.38	407.95	< 0.0001
B^2	1.52	1	1.52	21.17	0.0003
C^2	3.76	1	3.76	52.22	< 0.0001
Residual	1.22	17	0.072		
Lack of Fit	0.72	13	0.055	0.44	0.8848
Pure Error	0.51	4	0.13		
Cor Total	128.01	28			

Additionally, the PS, PDI, and EE% values showed small coefficients of variation of 0.61, 5.82 and 0.31, respectively, demonstrating the high trustworthiness of the experimental data. The R^2^ values for the coefficients of determination indicated that the models are able to justify, respectively, 98.76%, 98.37%, and 99.04% of the changeability in the responses.

The Adjusted R² of 0.9838, 0.9732 and 0.9842 agreed with the Predicted R² of 0.9745, 0.9541 and 0.9759 for the 3 models. Ultimately, the Adequate precision, or signals-to-noise ratios, for PS, PDI, and EE% were 46.90, 27.62, and 51.99, correspondingly..

The residuals appear to obey a normal plot, as shown by the linear shape, in the normal probability plots of the residuals for the 3 responses (Fig. S1). One helpful tool for figuring out the most appropriate power transformation is the Box-Cox plot. Our results (Fig. S2) demonstrate that the existing lambda (λ = 1) was sufficient for the 3 responses. A satisfactory agreement was shown between the predicted and observed data in all responses in the predicted against the actual plot (Fig. S3). The points were haphazardly dispersed around zero in the residuals vs. run number plot of all responses (Fig. S4), indicating that the models match our data.

**Fig. 1:**
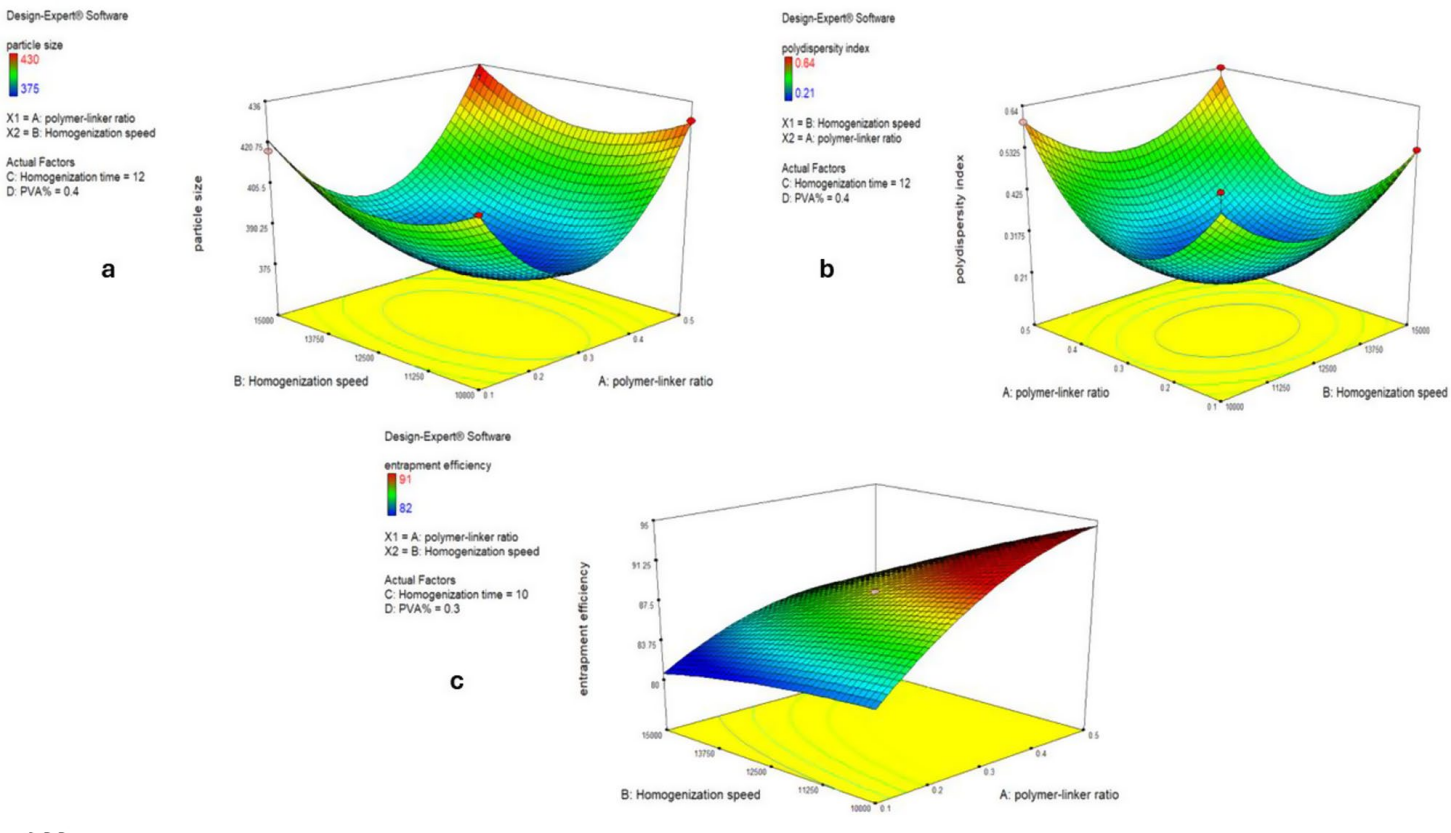
Three D model response surface analysis on NSs including the particle size (**a**), polydispersity index plot (**b**) and Entrapment efficiency (**c**)

Using the optimization function provided in the software, the polymer linker molar ratio of 1:3, the homogenization period of 10 min, the homogenization speed of 11,700 rpm, and the PVA% of 0.38 were suggested to be the optimal values for a minimal PS, a minimum PDI, and maximum EE%, as shown by the 3D plots in Fig. 1. After applying these suggested optimal conditions, the resulting optimized MICA-NS was characterized.

### Characterization of MICA-NS

**Fig. 2:**
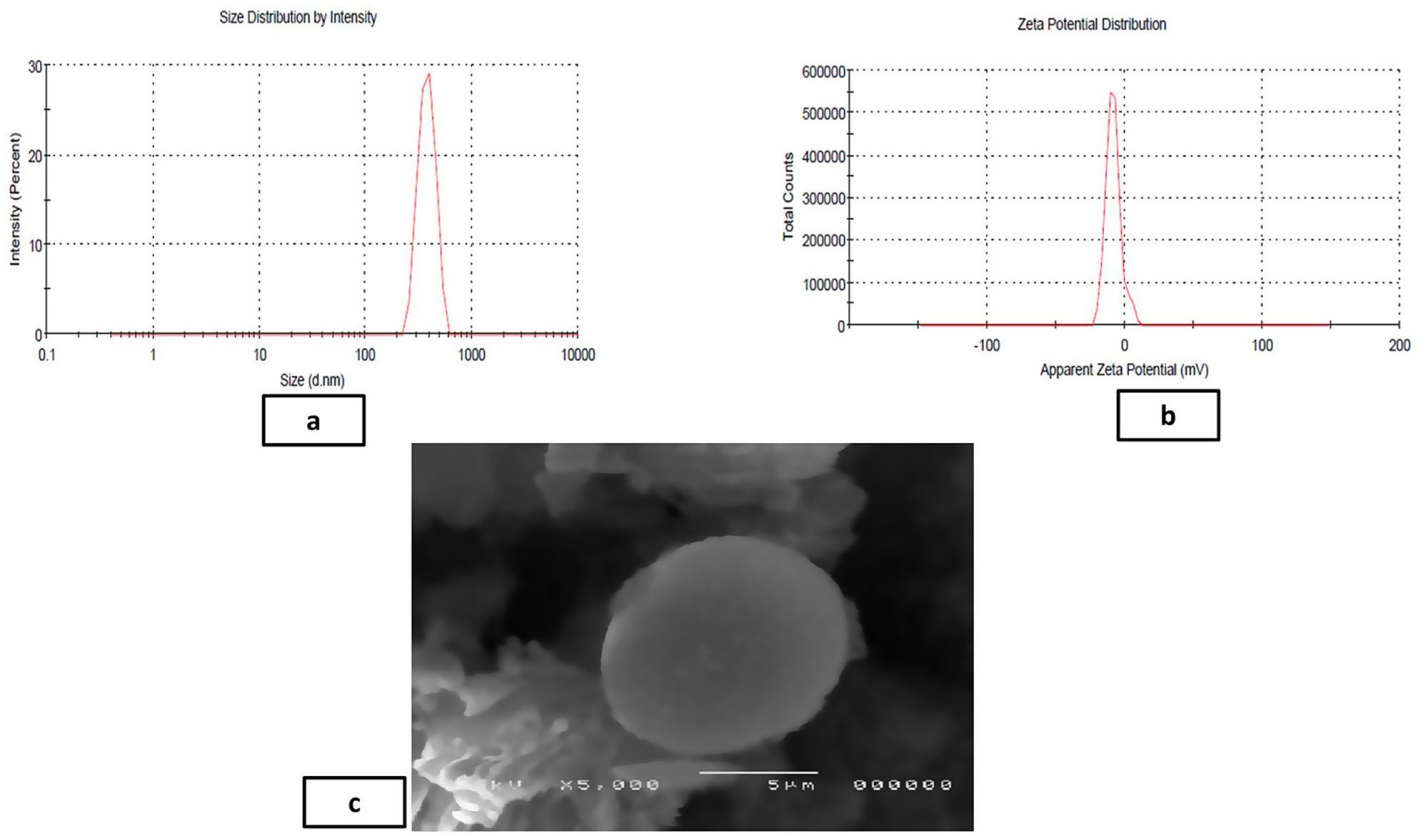
Analysis of the formulated optimized MICA-NS including particle size distribution (**a**), zeta potential (**b**) and SEM image (**c**)

The average yield of MICA-NS was 86.12%±0.81. The optimized MICA-NS had a PS of 378.33 ± 4.5 nm and a PDI of 0.15 ± 0.3. Additionally, a zeta potential of -17.8 ± 0.15 mV was measured. Figure 2a shows the size distribution by intensity for the optimal formula while Fig. 2b shows the dispersion of the zeta potential. The MICA concentration was calculated from the prepared standard curve [24] (Fig S5). The formula demonstrated an entrapment efficiency of 91.8 ± 0.44% and drug loading % (DL%) was found to be 89.32%±0.12. In Fig. 2c, the scanning electron microscopy (SEM) image of the NS reveals a porous surface and a spongy, nanosized spherical structure. The unique texture of the NS facilitates easier penetration of medication into its interpenetrating network.

### FTIR spectroscopy analysis results

The complex’s recorded FT-IR spectrum (Fig. S6a) is quite similar to the β-CD’s FTIR spectrum (Fig. S6b). This is because the chemical bonding characteristics of both are similar. Furthermore, the inclusion complex FTIR spectra revealed a narrowing of the wide β-CD hydroxyl band at 3370.72 cm^− 1^. The β-CD frequencies were determined to be 1029.24 cm^− 1^, 1157.84 cm^− 1^, and 2928.53 cm^− 1^. These match the symmetric and non-symmetric stretching of [C-C], [CH2], and the bending vibration of [O-H]. The disappearance of the distinctive peaks of MICA (mainly the aromatic C-H stretch at 3000–3100 cm⁻¹, C = O stretching (carbonyl group from carboxyl -COOH) at 1700–1720 cm⁻¹ and a sharp peak of Indole NH (-NH) stretch around 3300–3500 cm,⁻¹ (Fig. S6c)) in the complex indicates that these pharmacological sets are incorporated in the cavity.

### Differential scanning calorimetry indicators

**Fig. 3:**
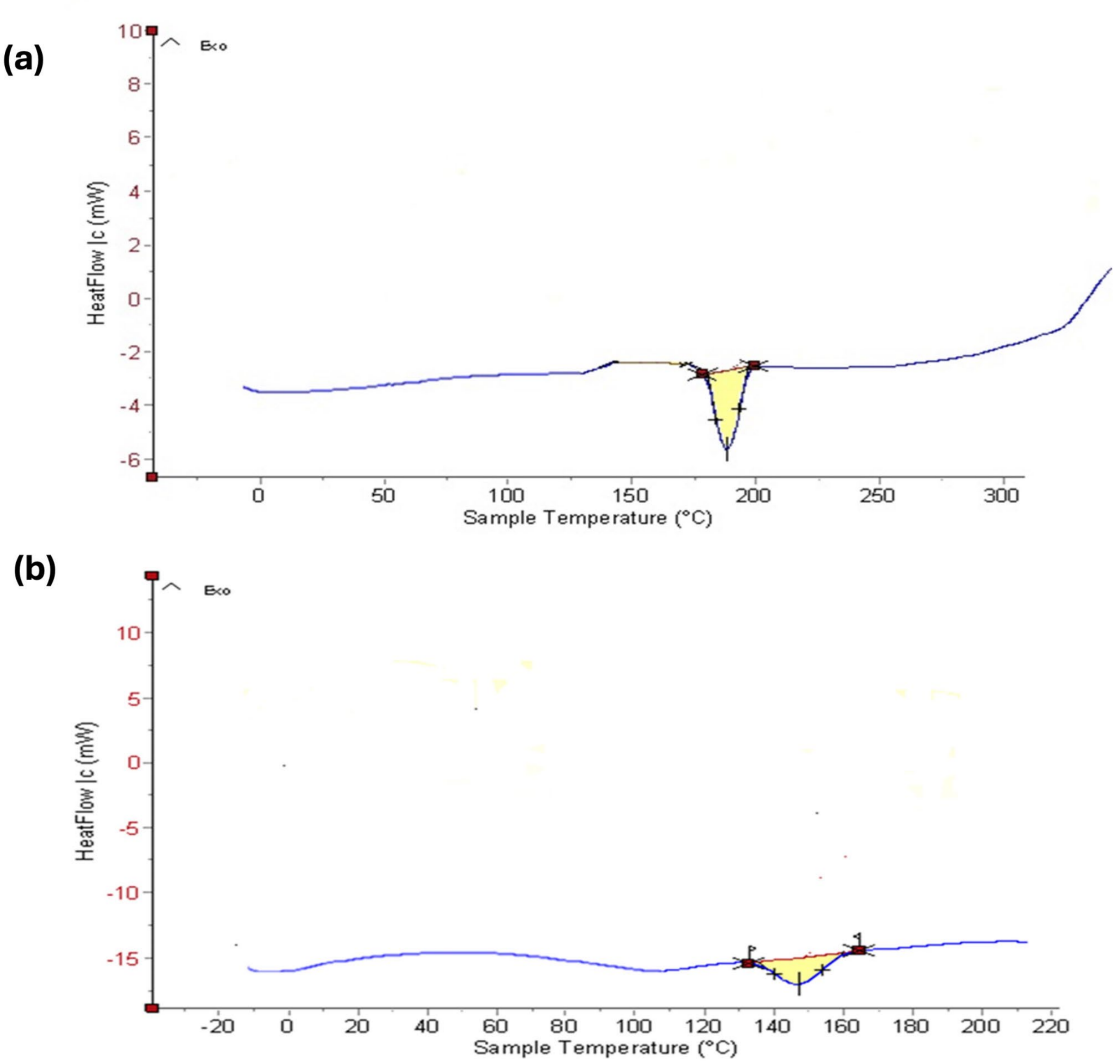
Differential scanning calorimetry thermograms of (**a**) pure MICA (**b**) optimized MICA-loaded nanosponge formulation

Figure 3a and b show the DSC thermograms for pure MICA and the optimized NS formulation, respectively. The melting point of MICA, which is represented by a strong peak on its endothermic DSC curve, is 195 °C. The drug encapsulation process of optimized MICA-NS results in a large endothermic peak on the DSC curve, which is created by the coalescence of β-CD and MICA in the porous cavities of NS. A stable, optimized MICA-loaded NS is shown by the removal of the abrupt drug peak and the upward line higher than the baseline (exothermic) curve, which indicates the blending of the drug and homogenous drug distribution in the polymer.

### Formulation and assessment of a topical hydrogel with an improved MICA-NS composition

For the MICA-NS-HG preparation, Carbopol 940 was ultimately selected as the optimal gelling agent based on its superior visual characteristics, as detailed in Table S1. Using carbopol 940, various gel formulas were created (Table S2). At a fixed concentration, the other elements’ compositions, however, did not change. Since formula HG-4 was the most superior, it was chosen for additional research. The findings for characteristics of the various preparations are shown in Table S3. Concerning the chosen gel’s characteristics, HG-4 displayed the most ideal measured values and had a translucent look with a silky and regular consistency. Skin compatibility was confirmed by the pH of 6.42 ± 0.28 and spreadability of 5.93 ± 0.41 cm. The extrudability was measured to be 1.25 ± 0.40 (g/s), and the swelling index was reported as 361.8 ± 0.22%. In addition, the predicted viscosity was 1088 ± 2 cps. The drug content percentage for the chosen hydrogel was 91.42 ± 0.56%, indicating that the drug was dispersed evenly throughout the HG. The drug content percentage for the chosen hydrogel was estimated using the standard curve (Fig. S5), and was found to be 91.42 ± 0.56%, indicating that the drug was dispersed evenly throughout the HG.

### In vitro release

**Fig. 4:**
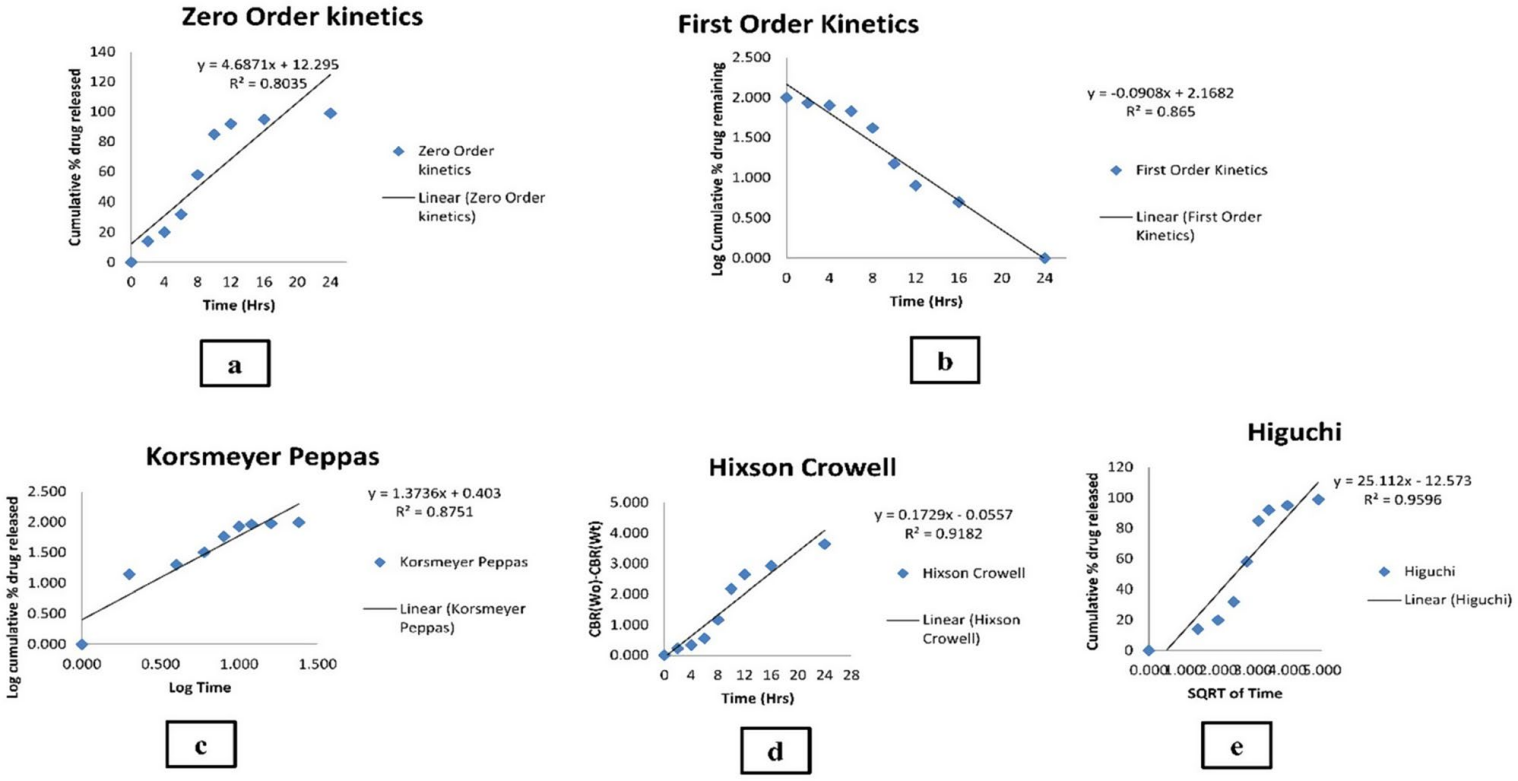
Various kinetic models of the in vitro release data (**a**) Zero order, (**b**) First order, (**c**) Korsmeyer and Peppas, (**d**) Hixson and Crowell model and (**e**) Higuchi ’ s model

The fabricated MICA-NS-HG subjected to in vitro drug release showed an initial burst release followed by sustained drug release. The initial burst effect was resultant to the desorption of drug on the NS surface. The porous matrix formed by polymer was conferred for sustained and progressive release of MICA (Fig. S7). The Higuchi model (R^2^ value = 0.9596) provided the greatest fit for the release. The Higuchi model was the best fit for the drug release data because it describes a diffusion-controlled release mechanism, which aligns well with the observed in vitro release profile of MICA-NS-HG. The model assumes that drug release occurs through a porous matrix system, where the drug diffuses out in a time-dependent manner. This is represented by the highest correlation coefficient (R^2^) of the Higuchi model among other kinetic models [48]. Utilizing the Korsmeyer-Peppas model, the MICA release mechanism from the produced formula was examined. Both the diffusion and polymer chain relaxation are responsible for monitoring drug release, as suggested by a non-fickian model (anomalous transport) with a diffusion exponent of 0.501. The in vitro release data’s several kinetic models are shown in Fig. 4. Table S4 compares the correlation coefficients of the various models that were used.

### Photodegradation analysis and stability results

Comparing the irradiation sample to the non-exposed samples, the irradiated sample’s drug content was 90.21 ± 31%; with a statistically insignificant difference (*p* > 0.05). The physical appearance, viscosity, pH, and drug content did not significantly alter after storage for three months at RT and in a refrigerator (*p* > 0.05) (Table S5).

### In vitro activity

In comparison to control fluconazole, (IZ = 24.4 ± 0.2 mm), MICA-NS-HG demonstrated improved in vitro antimycotic activity against *C. albicans* by a factor of 1.1 (IZ = 26.4 ± 0.5 mm).

### Cytotoxicity results

The optimized MICA-NS’s IC50 values, which indicate the 50% suppression of cell growth in vitro, were found to be 312.2 µg/mL which was higher than 287.8 µg/mL for fluconazole, the reference standard.

### In vivo studies

#### Survival rate study

The animals’ survival rates were 100% for the positive control groups F, G, and H, as well as in the treated groups A and E. In contrast, the same rate was found for control groups B and D (40%), and control group C had comparatively higher survival rates (60%).

#### Wound size measurement

**Fig. 5:**
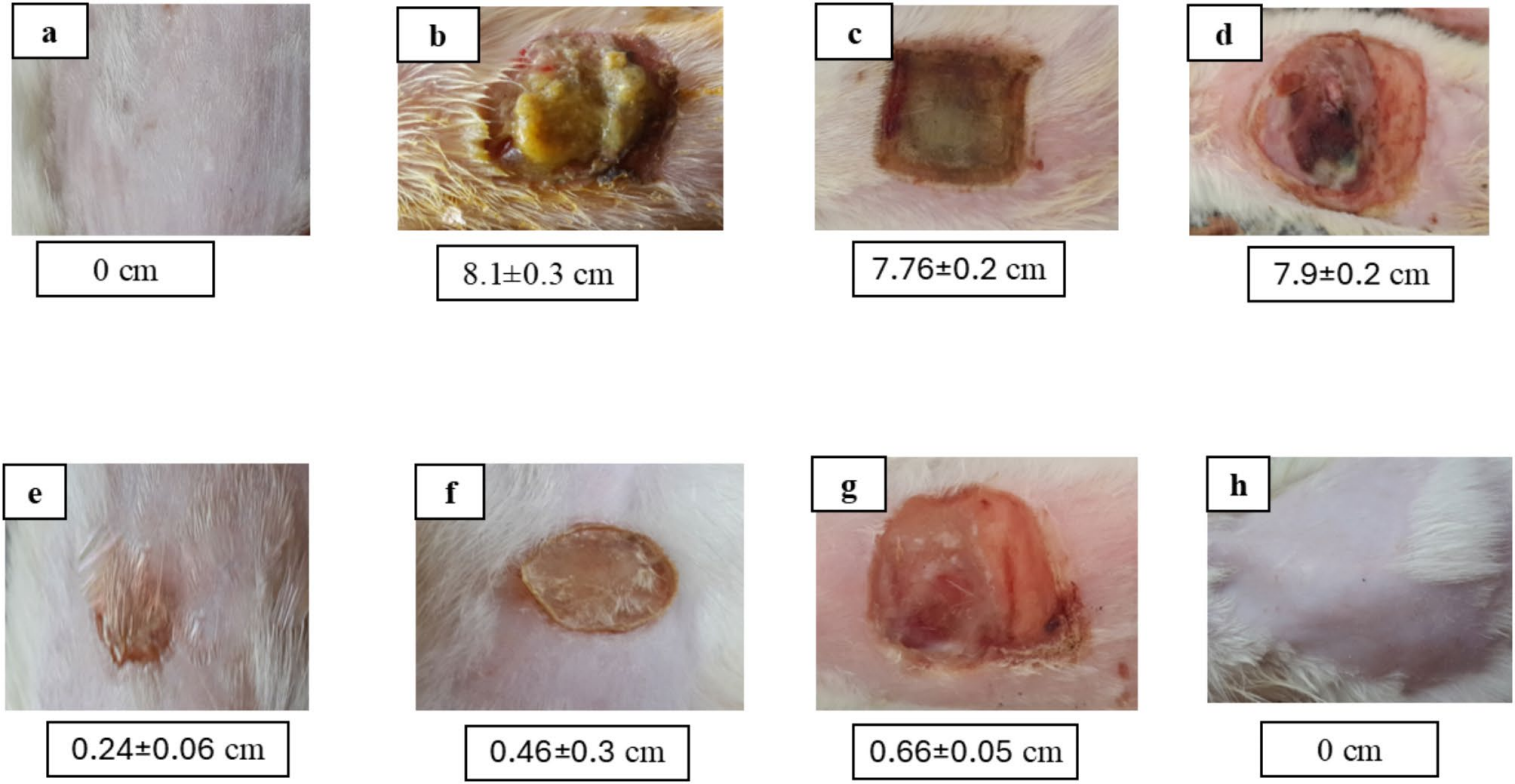
Mean wound size (cm) at 14th day of injury of a representative rat from each group as follows: (**a**) unburned, uninfected, treated with MICA -NS-HG (skin irritation test); (**b**) Control, burned, infected, untreated; (**c**) control, burned, uninfected, untreated; (**d**) control, burned, infected, treated with vehicle (negative control hydrogel); (**e**) burned, infected, treated with MICA -NS-HG; (**f**) positive control-1, burned, infected, treated with Collagenase 0.6 IU (Iruxol^®^, Abbott Co., Wiesbaden, Germany); (**g**) positive control-2, burned, infected, treated with Isoconazole hydrogel (ISN) 1% (Candicure^®^, Al-Esraa Pharmaceutical Optima Co., EGYPT); (**h**) normal control group (intact, unburned, uninfected, untreated)

Group E’s wound size contraction was measured at 76% on day 14. This was 1.4 times higher than positive control group F’s contraction of just 54% and 2.2 times more than group G’s contraction of 34%. Conversely, as the wound size increased, control groups B, C, and D displayed worsening wound contraction. Group E’s mean wound diameters differed from groups B, C, D, and G significantly (*p* < 0.05), but insignificantly from group F (*p* > 0.05). As seen in Fig. 5, data and photos related to wounds are displayed as Mean ± SD.

### Skin irritation study

The erythema and edema final observations received a score of zero after the application of our formula on the rat’s undamaged skin.

### Histopathological examination

**Fig. 6:**
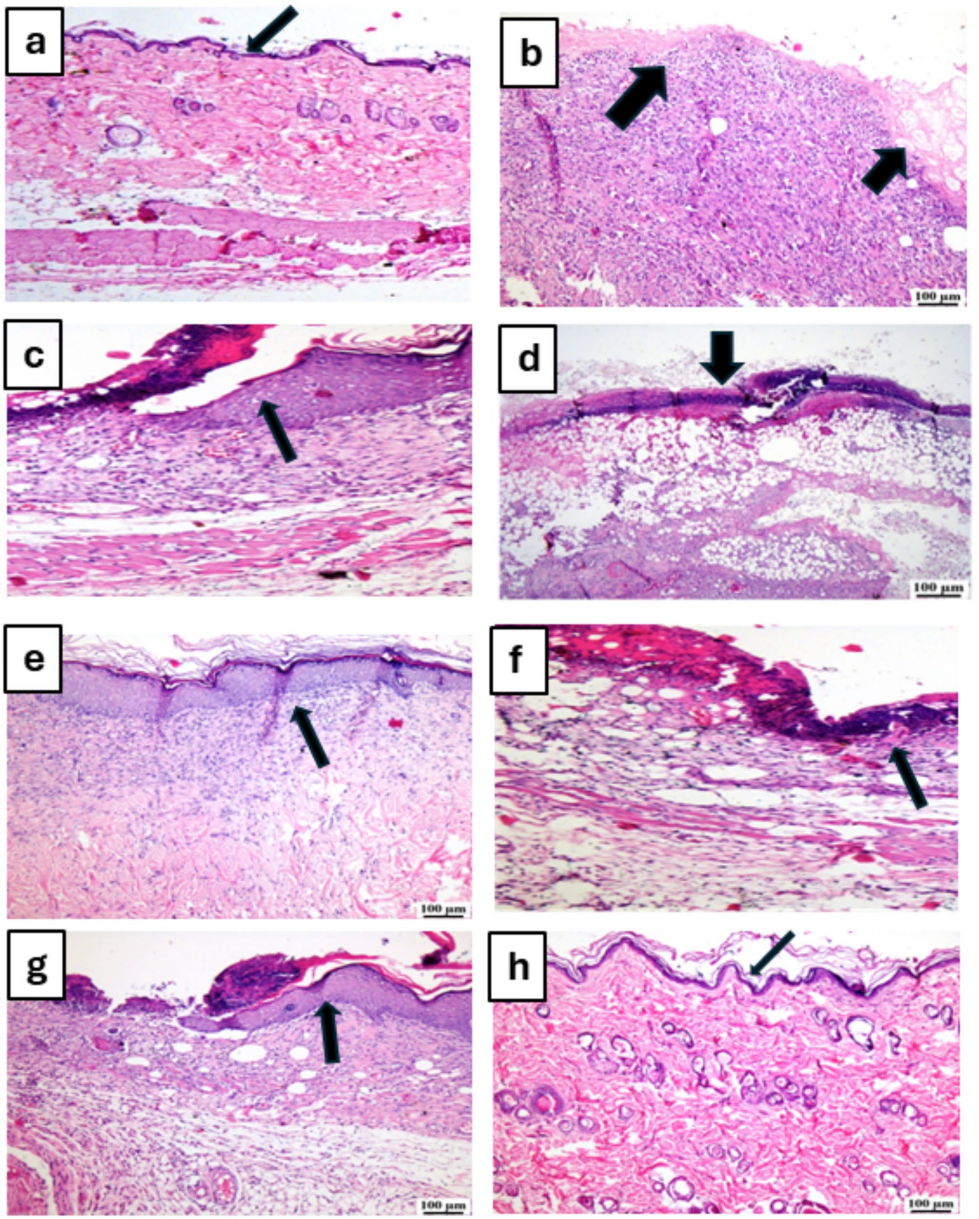
Photomicrographs of skin for hematoxylin and eosin (H&E) of the different tested groups as follows: (**a**) unburned, uninfected, treated with MICA -NS-HG (skin irritation test; (**b**) Control, burned, infected, untreated; (**c**) control, burned, uninfected, untreated; (**d**) control, burned, infected, treated with vehicle (negative control hydrogel); (**e**) burned, infected, treated with MICA -NS-HG; (**f**) positive control-1, burned, infected, treated with Collagenase 0.6 IU (Iruxol^®^, Abbott Co., Wiesbaden, Germany); (**g**) positive control-2, burned, infected, treated with Isoconazole hydrogel (ISN) 1% (Candicure^®^, Al-Esraa Pharmaceutical Optima Co., EGYPT); (**h**) normal control group (intact, unburned, uninfected, untreated). Arrows indicate significant and specific histopathological changes in the tissues of each group as indicated in the text

**Fig. 7:**
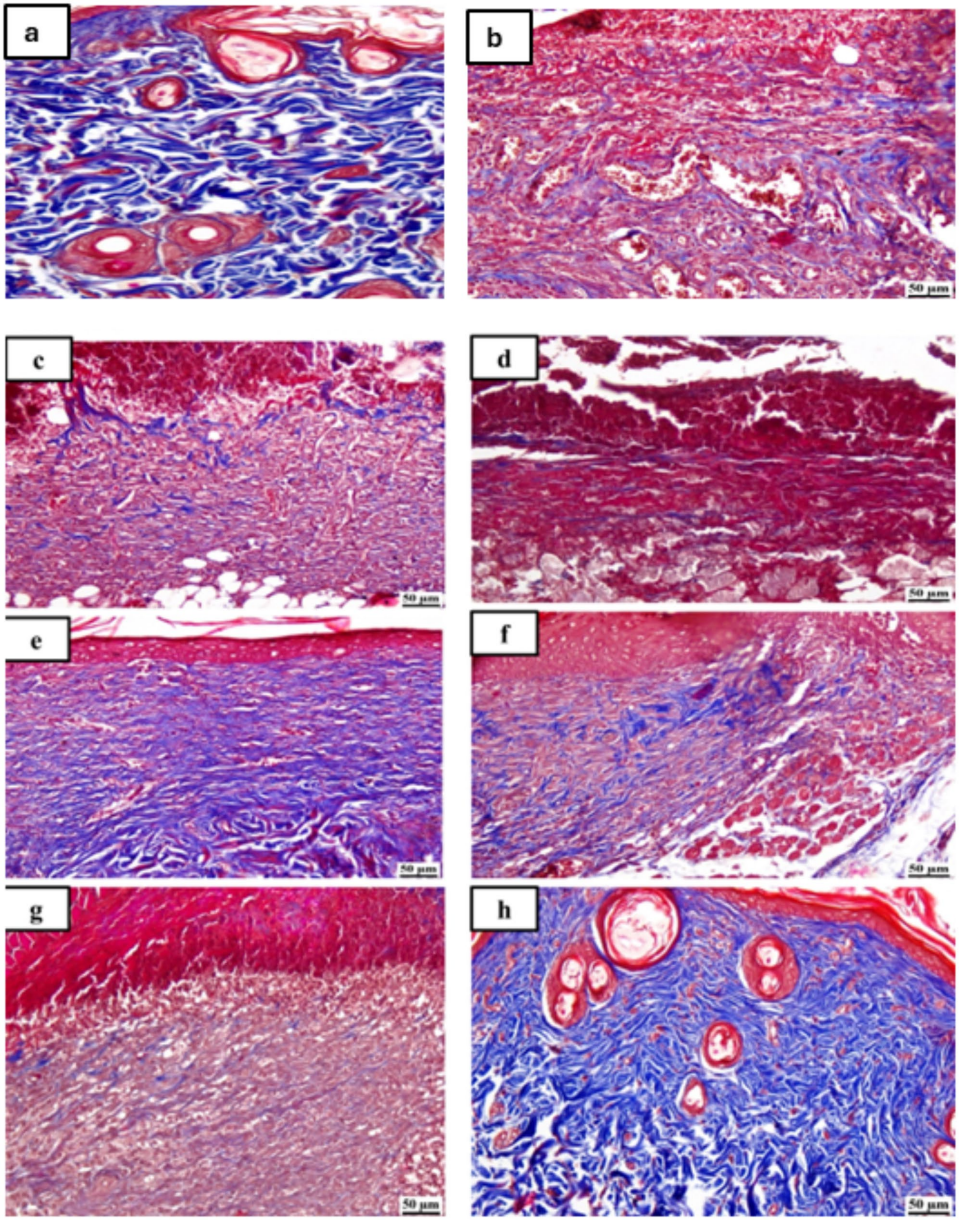
Photomicrographs of skin for Masson’s trichrome staining (MTC) of the different tested groups as follows: (**a**) unburned, uninfected, treated with MICA -NS-HG (skin irritation test; (**b**) Control, burned, infected, untreated; (**c**) control, burned, uninfected, untreated; (**d**) control, burned, infected, treated with vehicle (negative control hydrogel); (**e**) burned, infected, treated with MICA -NS-HG; (**f**) positive control-1, burned, infected, treated with Collagenase 0.6 IU (Iruxol^®^, Abbott Co., Wiesbaden, Germany); (**g**) positive control-2, burned, infected, treated with Isoconazole hydrogel (ISN) 1% (Candicure^®^, Al-Esraa Pharmaceutical Optima Co., EGYPT); (**h**) normal control group (intact, unburned, uninfected, untreated)

Figures 6 and 7 display photomicrographs that illustrate the histological characteristics of the skin layers and the healing manner in various groups.

Group (A): A skin photomicrograph showing the normal histological structures of the several skin layers, and integrated epithelium overlaying an intact epidermal layer (Fig. 6a). Furthermore, as observed in Masson’s trichrome-stained tissue segments of all the samples (MTC) (Fig. 7a), it showed significant regularly distributed collagen deposition up to 38.6% of the mean area percentage of the dermal material.

Group (B): exhibited a large amount of necrotic tissue debris and substantial cutaneous collagen necrosis; highly inflammatory granulation tissue filled the wound space (arrows). Additionally, inflammatory cell infiltrates and certain localized cutaneous hemorrhagic patches are visible (Fig. 6b). Additionally, less developed collagen fibers are seen, with an average dermal layer content area of 11.21% (Fig. 7b).

Group (C): showed epidermal hyperplasia at the edges of the wound and moderately inflammatory granulation tissues populating the gap with a crust of necrosis (Fig. 6c). Insignificant records of mature collagen were also shown by this group (Fig. 7c), reaching up to 17.31% of the mean area percentage of the dermal layer content, which resulted in reduced collagen deposition in the wound gap (MTC).

Group (D): showed results that were comparable to group B’s. Photomicrograph of skin displaying extensive dermal collagen necrosis and a buildup of necrotic tissue debris (arrows) in the wound gap filled by intensely inflammatory granulation tissue (Fig. 6d). Additionally, a photomicrograph of the skin (Fig. 7d) demonstrates limited collagen deposition up to 10.37% of the mean area percentage of the dermal layer content, in the wound gap (MTC). Photomicrograph of skin in group (E) demonstrates full re-epithelization and a higher, faster degree of wound gap healing, indicating hyperplastic epidermal remodeling and collagen-rich filled granulation tissue. Furthermore, Fig. 6e) shows increased fibroblastic activity together with a noticeable decrease in inflammatory cells (arrows). Figure 7e also indicated increased records of dermal collagen fibers up to 32.05% (almost three times more than in Group B).

Group (F): minimal granulation tissue inflammation was revealed and continued documentation of a small ulcerated wound gap that concealed necrotic tissue debris (Fig. 6f). Dermal collagen fiber maturation was modest (Fig. 7f) and reached 21.65%.

Group (G): mildly inflammatory granulation tissue filling the wound gap, with epidermal remodeling beneath a necrotic crust at the wound’s edge (Fig. 6g); also, the collagen fibers in this group exhibit intermediate maturation, reaching up to 22.64%.

Group (H): showed the same records as group A, showing strong regularly distributed collagen deposition (Fig. 7h) up to 39.3% and an intact dermal layer and overlying epithelium (Fig. 6h).

### Ex vivo analysis

Results showed that the drug amounts that permeated from MICA hydrogel and isoconazole 1% (Candicure^®^) were 91.98 ± 5 and 60.041 ± 3 µg/cm^2^, respectively. This means that the drug that permeated from our hydrogel was 1.5 times more than isoconazole 1% (*p* < 0.05).

### Angiogenesis, inflammatory markers and Proinflammatory cytokines measurements

Group E’s mean COX-2, TNF-α, NF-kB -p105, IL-6, and IL-1β levels were, respectively, 3.3 ± 0.5 ng/mL, 56.760 ± 3 pg/mL, 16.259 ± 5 ng/mL, 58.560 ± 4 pg/mL, and 159.8 ± 2 pg/mL. These values were significantly lower than those of groups C, B, and D (*p* < 0.001), positive control groups G, and F (*p* < 0.05), and insignificantly different from values of groups A and H (*p* > 0.05). As determined by the VEGF levels assay, group E’s level of vascularization was found to be significantly higher (362.157 ± 4 pg/mL) than that of groups C, B, and D (*p* < 0.001), four times greater than that of positive control F (86.528 ± 6 pg/mL), five times higher than that of positive control G (69.356 ± 5) (*p* < 0.05), and insignificantly different from that of groups A and H (*p* > 0.05).

## Discussion

Our study aimed to develop a topical formulation of MICA, that would meet al.l the requirements for topical treatment. In our earlier study, MICA, a strong antifungal agent, from *B. toyonensis* isolate OQ071612 culture broth was isolated and chemically characterized [24]. In response to the growing demand for natural remedies, sustainable fermentation technologies are of particular concern globally [49]. As a result of rising antibiotic resistance and overuse, naturally occurring antimicrobials must be used in place of chemically synthesized ones [50]. Scientists are striving for nanocarriers with unique characteristics, maximum efficacy, less adverse effects, and specificity. The NS particles offer several advantageous properties, including water-solubility, water-stability, hydrophobic drug-enclosing, targeted drug delivery, low toxic side effects, stability over a pH 1–11, thermostability till 130 °C, flavor masking capabilities, biodegradability, and the ability to convert liquids into solids. These combined features make NS particles an effective solution for various applications [11, 51]. Furthermore, the cross-linker to-polymer ratio can be changed to make particles larger or smaller, and their drug release can be controlled [11, 34].

Because bacteria cannot pass through the 0.25-millimeter pores of NSs, they are also self-sterilizing [11]. Moreover, the NS have been used against SARS-CoV-2 to limit fungal co-infections [52]. Additionally, according to Wang et al., these cellular NSs could be helpful for biologically neutralizing pathogenic antibodies, bacterial toxins, inflammatory cytokines, chemical hazardous agents, and virus fragments [51]. Increased entrapment, which helps NSs function as a reservoir for various pharmacological compounds, is another advantage of employing NSs. Furthermore, they help shield compounds from deterioration [13].

Therefore, the emulsion-solvent diffusion approach was employed to create NSs, which were then used to deliver MICA. This approach has two benefits: it does not need emulsifiers, and it can be prepared simply and quickly while producing meaningful outcomes [52]. Moreover, it enhances drug entrapment within polymeric carriers, reducing drug loss during preparation, and is suitable for hydrophobic and hydrophilic drugs [53]. This technique also provides more encapsulation capacity compared to other methods of manufacturing with no requirement for homogenization. It also gives higher batch-to-batch reproducibility with ease of scale-up, simplicity and narrow size distribution. Moreover, it allows a precise control of particle size that is difficult with other usual method of preparation of nanoparticles like nano-precipitation [54]. To optimize the process parameters and produce a cost-effective outcome, we employed the RSM, more especially the Box-Behnken design (BBD) [14]. In earlier research, this design was effectively used to maximize the production of antibiotics [55], and formulate secondary antifungal metabolites encapsulated in NSs [31].

The concentration of entrapped drug can be calculated using the method described by Fahmy et al. [56]. However, an indirect method of measuring entrapment efficiency involves measuring the concentration of unentrapped drugs using spectrophotometry at λmax. As an alternative, EE can be determined directly by breaking the developed nanoparticles’ membranes as previously mentioned [31]. However, the indirect approaches could only be utilized throughout the preparation phase and not after freeze-drying [57]. According to prior research, the homogenization time and speed significantly boost the entrapment efficiency [31]. Reduced entrapment efficiency was associated with lower crosslinker concentrations, which is consistent with earlier findings [28]. However, intermolecular hydrogen bonds could be damaged by diphenyl carbonate [58]. Therefore, it was determined that process parameter optimization was required to reach an ideal proportion between β-CD and DPC. In line with other research, PS, PDI, EE% were regarded as very important parameters and had an impact on the creation of NS [28].

Based on our findings, the optimized MICA-NS was determined to have a particle size of 378.33 ± 4.5 nm. The scanning electron microscopy (SEM) analysis confirmed their homogeneous nature, porous surface, spherical form, and drug-containing polymer matrix. SEM is a reliable method for revealing a material’s morphology, providing valuable data on pore structure and overall homogeneity [37]. Zeta potential is the main marker for the colloidal dispersion’s stability. By including a zeta sizer or an extra electrode in the particle size analysis apparatus, the zeta potential can be ascertained. The dispersion quality and stability of MICA-NS are comparatively good, as evidenced by its average ZP of -17.8 ± 0.15 mV [33]. This is consistent with research that found similar negative ZPs for NSs and that generated stable water suspensions that do not aggregate with time [30, 59]. A colloidal dispersion’s ZP, whether positive or negative, increases with stability [13]. When it comes to particle size distribution characterization, the PDI is a metric that is used to define the size range of the NS systems. “Polydispersity” refers to the extent of non-uniformity in a PS [30]. Values less than 0.05 are frequently only encountered with exceptionally monodisperse standards due to the scale of this metric [60]. With a PDI of 0.15 ± 0.3, MICA-NS exhibits a decent and satisfactory monodisperse system. Dynamic light scattering (DLS) analysis is probably not acceptable for a sample with a very large PS distribution and a PDI > 0.7 [61].

The entrapment efficiency was shown to be within an appropriate range of 91.8 ± 0.44%, confirming findings from a previous study conducted by Amer et al. [62]. The method of drug packing into the NS can affect the drug-NS complexation. Freeze drying has been shown to modify drug and NS complexation in certain circumstances, even though the effectiveness of an approach is primarily dictated by the characteristics of the drug and polymer [13]. According to numerous studies, CD-based NP may sustain a high loading capacity of different small molecules for drug delivery. This is supported by the high drug loading capacity that MICA-NS formulation has reached [11]. According to Tejashri et al., NSs are the best option for addressing problems including solubility, delayed release, and active drug stability since they possess a greater loading capacity than other nanocarriers [63].

Differential scanning calorimetry (DSC) measures the heat absorbed or emitted during heating or cooling processes [64]. It is utilized to assess the reaction heat, heat capacity, melting point and glass transition [64]. DSC of optimized MICA-NS confirmed its development, resulting in a decrease in the drug’s crystallinity. The shift from a crystalline to an amorphous structure validated the formation of the NS [65]. The peak broadens due to the molecularly scattered phase of MICA within the NS structure. Overall, the amorphous structure of the MICA-NS enhances drug entrapment within the NS [33].

A number of gelling agents have undergone preliminary testing in order to prepare the hydrogel, with carbopol 940 being chosen. After encountering triethanolamine, the hydrophilic polyacrylic acid polymer, carbopol 940 ionizes its carboxyl functional groups. Because charged polymer strands repel one another electrostatically, a gel-like structure is formed as a result [66]. At this point, the produced HG’s pH is raised to a level appropriate for topical administration. Moreover, carbopol can be utilized in gel compositions due to its non-toxicity and non-irritating properties [67]. The benefit of Carbopol is that it may be created in room temperature water, in contrast to hydroxypropyl methylcellulose, which needs to be manufactured in hot water. Moreover, carbopol 940 was chosen for hydrogel preparations due to its broad viscosity range [66]. The viscosity of the preparation is directly influenced by the carbopol 940 concentration, which also affects its physical features [68, 69]. For a gel composition to be accepted, good spreadability is essential. Our findings revealed that the hydrogel (HG) had a pH of 6.42 ± 0.28 and a spreadability of 5.93 ± 0.41 cm. The neutral pH of the hydrogel suggests a low likelihood of skin irritation [70].

The viscoelastic and mechanical characteristics, degradation rate, degree of crosslinking, and refractive index can all be ascertained using the swelling data [68, 69]. The gel showed good swelling characteristics that are consistent with earlier findings, with a favorable SW% of 361.8 ± 0.22% [71]. The produced hydrogel had a good consistency and texture, as evidenced by its viscosity of 1088 ± 2cps, while its extrudability was measured at 1.25 ± 0.40 (g/s). In general, the consistency of hydrogel formulations is reflected in their viscosity [71, 72].

The drug content percentage for the chosen hydrogel was 91.42 ± 0.56%, indicating a high level of drug retention by the NS system. The Higuchi diffusion kinetic model provided the best match for the release, as indicated by the coefficient of correlation (R^2^ value = 0.9596). This model is predicated on multiple conjectures, such as the following: drug diffusivity is constant, matrix swelling and dissolution are negligible, and the initial concentration of the drug in the matrix is significantly greater than drug solubility [73]. The process of MICA release from the generated formula was examined using the Korsmeyer-Peppas model. Diffusion and polymer chain relaxation work together to influence release in a non-fickian model (anomalous transport) represented by a diffusion exponent (*n* = 0.501) [7]. The medication amount was reevaluated after being exposed to UV light and after stability tests were carried out in the given circumstances [7]. The remarkable stability characteristics of the formulation were demonstrated by the results, which showed that there was an insignificant difference (*p* > 0.05) when compared to the initial conditions. An in vitro antifungal investigation showed that MICA-NS-HG had better antifungal activity than fluconazole, the positive control, against *C. albicans* ATCC10231.

The MTT assay, which was first used by Mosmann in the 1980s, has evolved into the industry as typical for assessing cell cytotoxicity [74]. The optimal MICA-NS and fluconazole had IC50 values of 312.2 µg/mL and 287.8 µg/mL, respectively. Both samples decreased cell viability in a manner that was dependent on the dose [75]. Fluconazole, a water-soluble bis-triazole, is widely used to treat candidiasis, cryptococcosis, and endemic mycoses due to its high tolerance, low toxicity, and favorable pharmacokinetics [76]. Resistance often arises from the replacement of susceptible *C. albicans* with less sensitive species like *C. glabrata* and *C. krusei*. Fluconazole was considered as a control, being a core antifungal agent in the treatment of systemic and superficial fungal infections, particularly due to its broad activity against *Candida* species. Lastly, to evaluate the MICA-NS hydrogels’ ability to inhibit the pathogenicity of *C. albicans* in rats, a model of thermal damage in male Wistar albino rats contaminated with the pathogen was created. Numerous studies have previously reported that mice or rats with superficial skin infections were caused by *C. albicans* [77, 78]. Isoconazole(ISN) is commonly used in topical formulations for dermatophytic infections, making it relevant for comparing topical delivery systems like the nanosponge-hydrogel, so it was used as a positive control. Isoconazole, an azole antifungal, is primarily used for superficial fungal infections caused by dermatophytes (~ 70%), yeasts (~ 35%), and non-dermatophytic molds [79]. Our findings showed that, in comparison to other control groups, our formulation boosted both the survival rate, and the amount of dermal collagen fibers deposited in the treated groups skin. It was suggested in a different study that β-CD could increase the efficacy of antifungal drugs in the form of NSs. This is because β-CD interacts with *C. albicans* to create changes to the cell wall and to interfere with its protective function. In addition, *C. albicans* may be more resistant to larger concentrations of β-CD than other microorganisms because glucosylceramides found in its cell wall trigger an internal signaling mechanism that causes the fungal pathogen to undergo apoptosis. This eliminates the need for synthetic and semi-synthetic drug side effects when using antifungal medicines manufactured as β -CD-NS for highly resistant fungal infections [59]. According to Behere and Ingavle, the healing process requires chronological steps to restore cells to their pre-injury state [80]. Fungal infection can also cause a delay in the healing process. To evaluate the MICA-NS-HG’s capacity to heal wounds, the area of the wound and the proportion of wound contraction were assessed. Days 1, 7, and 14 after the wound was formed were used to measure the wound areas and wound contraction (%) in the current study. The study’s findings suggest that MICA-NS-HG can be used to treat and hasten the healing of wounds infected with *C. albicans*. MICA-NS-HG treated group had a 76% wound contraction proportion, which was 1.4 times higher than group (F) and 2.2 times greater than group (G). We believe that this is the first study to look at the effectiveness of MICA-NS-HG in treating candidiasis in a model of thermal damage in rats. Different wounded groups’ histopathological examinations showed differing levels of tissue damage and recovery processes. Because isoconazole and collagenase have wound-healing qualities, we utilized them as positive controls in this research, as previously reported [31]. Topical application of MICA hydrogel in group (E) demonstrated a significant difference (*p* < 0.05) in reducing local wound infection and boosting skin rejuvenation compared to the control group B (burned, infected, untreated). Additionally, there was a significant hastening of dermal mature collagen fiber development. Therefore, our results corroborate a prior study by Srivastava et al. suggesting that antimycotic therapy NSs could be effective in counteracting *C. albicans* infections linked to wounds [28]. The high drug quantity in the in vitro penetration investigation validated the topical gel formulation’s effective dermal administration of MICA-NS via skin.

Molds, yeasts, and dermatophytes are a few of the pathogens that can cause dermatomycoses, or superficial fungal skin infections [7]. Inflammation, pruritus and erythema, are quite common due to fungal exo-enzymes [81]. Therefore, it was decided that an ELISA technique would be required to quantify the levels of angiogenesis (VEGF), inflammatory indicators such as COX-2 and proinflammatory cytokines. Angiogenesis is essential for the healing of wounds (Ahmad and Nawaz 2022). Vascular endothelial growth factor (VEGF) is the most important and thoroughly studied angiogenic factor [82]. According to many experts, VEGF stimulates collagen deposition and wound epithelialization, resulting in the production of high amounts of VEGF during typical wound healing [83]. According to earlier results, the MICA hydrogel-treated group (E) showed considerably (*p* ˂0.05) greater VEGF levels than the infected untreated group (B), indicating improved levels of re-epithelization and collagen deposition [31].

Enzymes known as cyclooxygenases are necessary for the synthesis of PGs from arachidonic acid. Injured tissues stimulate COX-2, which causes inflammatory processes, whereas COX-1 is constitutively expressed [84]. Anti-inflammatory effects could arise from COX-2 inhibition [84]. When compared to the infected untreated group (B), our formulation dramatically reduced the levels of COX-2, suggesting that it also had an additional anti-inflammatory impact [85].

Tumor necrosis factor (TNF)-α is quickly manufactured in the wounded area, inflaming the tissues surrounding the lesion [86]. Therefore, TNF-α was markedly neutralized in group E that received MICA-NS-HG treatment, showing that the wounded area had healed well, but it was raised in the infected untreated groups. The traditional NF-kB pathway is triggered during wound healing, which causes a multitude of cytokines, secondary inflammatory mediators, and apoptotic inhibitors to be produced [87]. Conversely, as previously demonstrated, overexpression of NF-κB can result in decreased wound healing [88]. In light of these details, the formulation demonstrated lower (*p* ˂0.05) NF-kB levels, indicating wound healing, in comparison to other untreated groups that displayed greater levels, indicating that the wound was still healing.

According to earlier research, IL-6 may have a part in the healing process [89]. Group E’s lower IL-6 levels (p ^<0.05) in comparison to untreated groups suggest a typical wound healing process. This aligns with the findings from previous studies [90]. IL-1β is an antagonist of extracellular matrix metabolism because it stimulates the proliferation of fibroblasts, encourages the synthesis of collagenase, and prevents the formation of endothelial cells [85]. Moreover, it has been demonstrated that IL-1β stimulates smooth muscle cell growth and attracts neutrophils and macrophages with its chemo-attractant properties [91]. The ELISA results of our investigation showed that the untreated groups had greater levels of IL-1β than the treated groups did. The results of Gürgen et al. and Aneesha et al. are consistent with this, suggesting that MICA hydrogel administration is efficient at suppressing inflammation via IL-1β [92]. The significant decreases in pro-inflammatory cytokines and COX-2 observed in group E suggest that MICA hydrogel accelerates healing by preventing inflammation. Finally, our study shows that the antifungal MICA can be effectively optimized for the manufacture of NSs. While this study demonstrates significant potential, several limitations must be considered. Although in vitro studies offer valuable insights into drug release kinetics and swelling behavior, they do not fully replicate in vivo physiological conditions. Factors such as enzymatic activity, pH variations, and complex biological interactions may influence the formulation’s performance. Additionally, the study does not provide comprehensive long-term stability data, which is essential for determining the shelf life and structural integrity of MICA-NS-HG formulations over extended periods. Furthermore, as the study is primarily limited to preclinical and in vitro evaluations, further validation through human clinical trials is necessary to assess therapeutic efficacy and ensure patient safety.

## Conclusion

In this study, MICA was successfully formulated into an optimized nanosponge preparation. The particle size analysis, assessed polydispersity index, measured zeta potential, and performed Fourier-transform infrared analysis on the generated NS resulted in an optimum topical hydrogel formula. The hydrogel exhibited favorable properties such as swelling, extrudability, and spreadability. Its neutral pH confirmed its safety for skin contact, and it contained a high drug content with potent antimycotic effects in vitro. Our investigation of in vitro drug release closely aligned with Higuchi’s concept. Stability studies further supported the robustness of this formulation. Subsequent in vitro, ex vivo, and in vivo studies demonstrated that the hydrogel effectively suppressed fungal growth and exhibited enhanced penetrability when applied topically. Moreover, the synthesized MICA-NS-HG reduced levels of several inflammatory cytokines in the treated group, mitigating inflammation and suppressing the illness. Additionally, compared to untreated groups, treated group E exhibited significantly higher VEGF levels, indicating improved vascularization. The MICA-NS-HG formulation developed in this study exhibits significant potential for clinical application in the treatment of superficial mycotic infections in humans. To further advance its therapeutic utility, phase I clinical trials are essential to assess the safety, tolerability, and preliminary efficacy of MICA-NS-HG in humans before advancing to larger-scale trials. Future clinical research should focus on evaluating the formulation’s therapeutic potential against *Candida albicans*-related topical infections, ensuring its effectiveness and safety for clinical translation and widespread medical use.

## Electronic supplementary material

Below is the link to the electronic supplementary material.

Supplementary Material 1

## Data Availability

The authors declare that the data supporting the findings of this study are available within the article and its supplementary information file. The 16 S ribosomal RNA of Bacillus toyonensis isolate OQ071612 deposited into the NCBI GenBank under the accession number OQ071612 (https://www.ncbi.nlm.nih.gov/nuccore/OQ071612).
