# Supplementary material for: In vitro and preclinical evaluation of the antifungal activity of 6-methoxy-1 H-indole-2-carboxylic acid produced by Bacillus toyonensis strain OQ071612 formulated as nanosponge hydrogel
Source: Microb Cell Fact. 2025 Apr 1;24:77. doi: 10.1186/s12934-025-02688-y (PMC11959791; doi:10.1186/s12934-025-02688-y)
Supplement: Supplementary file 1 — Supplementary Material 1 [file 12934_2025_2688_MOESM1_ESM.docx]

**In vitro and preclinical evaluation of the antifungal activity of 6-methoxy-1H-indole-2-carboxylic acid produced by *Bacillus toyonensis* strain OQ071612** **formulated as nanosponge hydrogel**

Sayed E. El-Sayed^1^, Neveen A. Abdelaziz^2,^ Ghadir S. El-Housseiny^3^, Khaled M. Aboshanab^3,4^*

^1^Department of Microbiology and Immunology, Faculty of Pharmacy, Ahram Canadian University, Sixth of October City, Giza 12451, Egypt

^2^Department of Microbiology and Immunology, Faculty of Pharmacy, Ain Shams University, Cairo 11566, Egypt

* **Corresponding Author: Khaled M. Aboshanab**

Address: Department of Microbiology and Immunology, Faculty of Pharmacy, Ain Shams University, Cairo 11566, Egypt.

E-mail:  [aboshanab2012@pharma.asu.edu.eg](mailto:ghadir.elhossaieny@pharma.asu.edu.eg)

Mobile: +201-0075-82620

Fax: (202)24051107

<https://orchid.org/0000-0002-7608-850X>

**Authors’ e-mails**

Sayed E. El-Sayed: [sayed.emad@acu.edu.eg](mailto:sayed.emad@acu.edu.eg)

Khaled M. Aboshanab:  [aboshanab2012@pharma.asu.edu.eg](mailto:ghadir.elhossaieny@pharma.asu.edu.eg)

Neveen A. Abdelaziz: [neveen.abdelaziz@acu.edu.eg](mailto:neveen.abdelaziz@acu.edu.eg)

Ghadir S. El-Housseiny: [ghadir.elhossaieny@pharma.asu.edu.eg](mailto:aboshanab2012@pharma.asu.edu.eg)


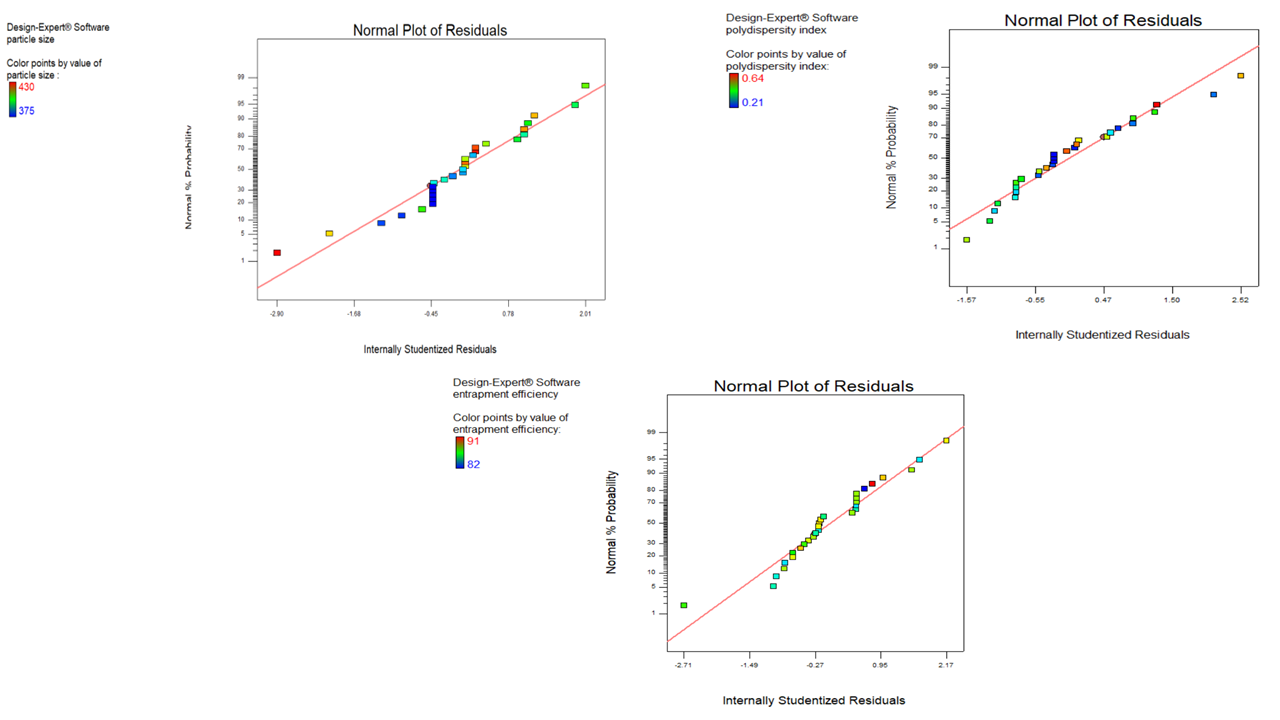


**c**

**b**

**a**

**Fig. S1** The normal probability plots of residuals for (a) Particle size (PS), (b) Polydispersity index (PDI), and (c) Percentage of entrapment efficiency (EE%)


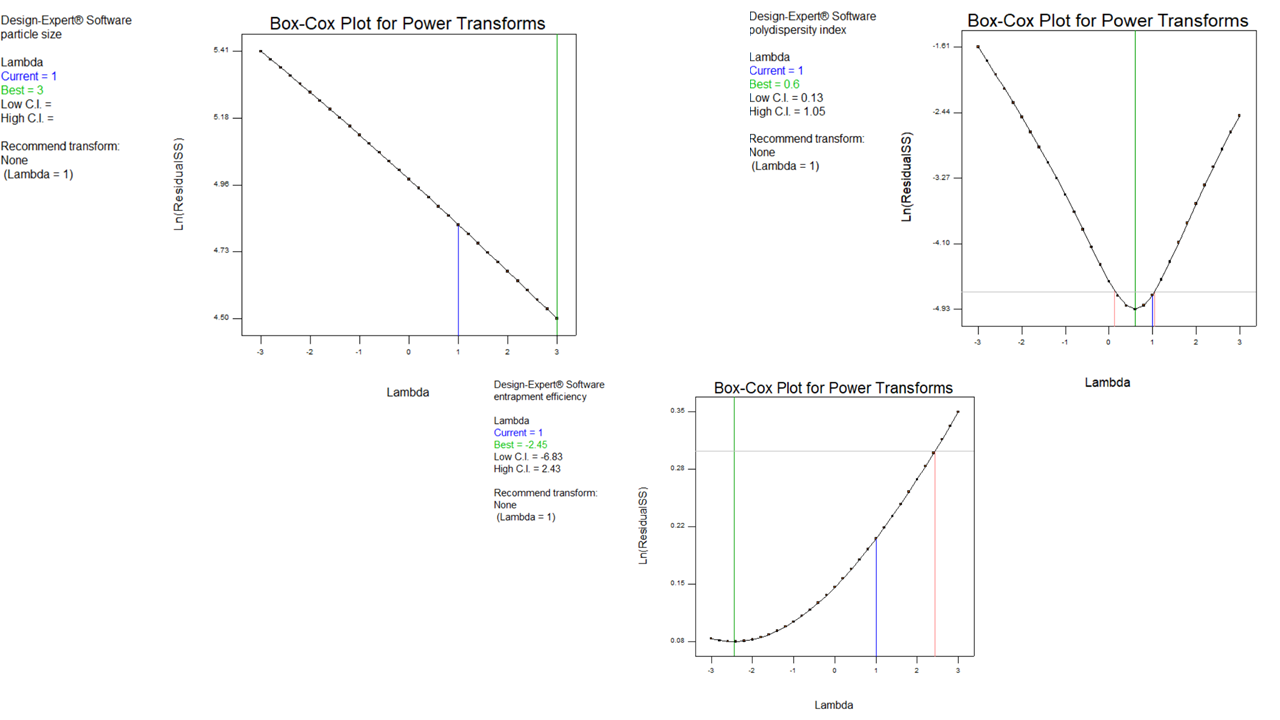


**c**

**b**

**a**

**Fig. S2** The Box-Cox plots of (a) Particle size (PS), (b) Polydispersity index (PDI), and (c) Percentage of entrapment efficiency (EE%)


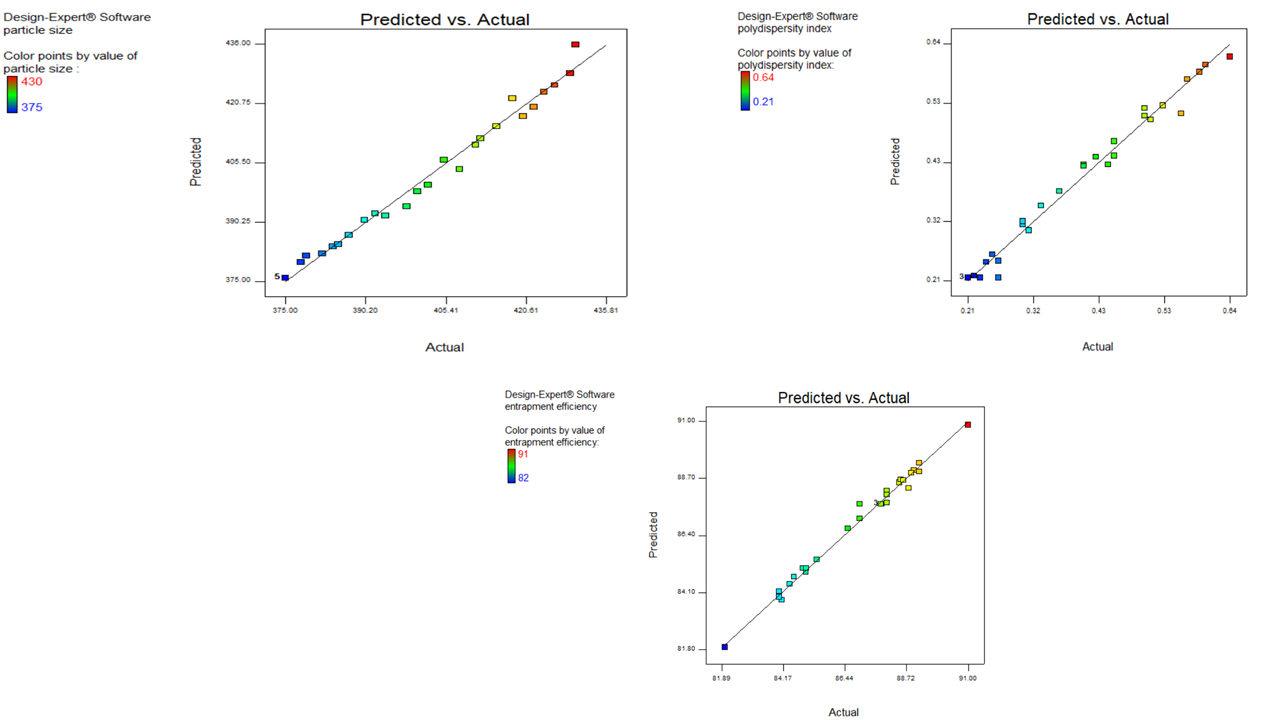


**c**

**b**

**a**

**Fig. S3** The predicted versus actual values plot of (a) Particle size (PS), (b) Polydispersity index (PDI), and (c) Percentage of entrapment efficiency (EE%)


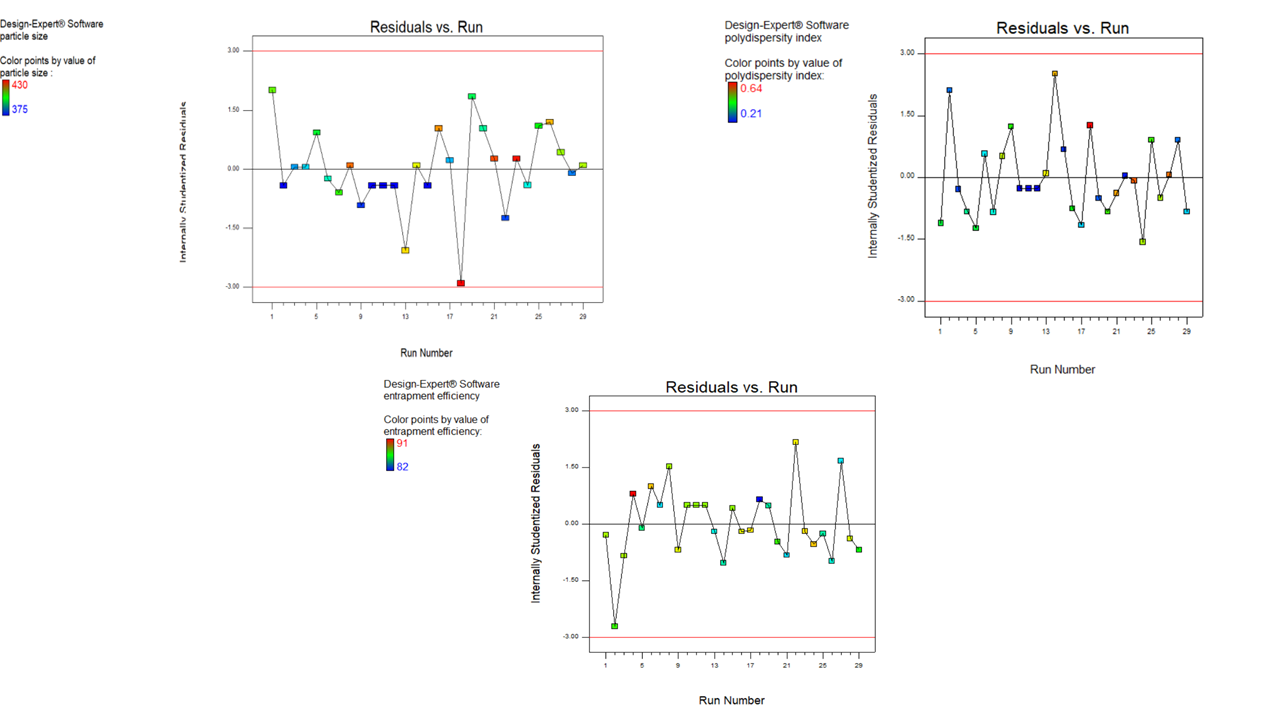


**c**

**b**

**a**

**Fig. S4** The residuals versus Run number plot of (a) Particle size (PS), (b) Polydispersity index (PDI), and (c) Percentage of entrapment efficiency (EE%)

**Fig. S5** Standard curve of MICA.


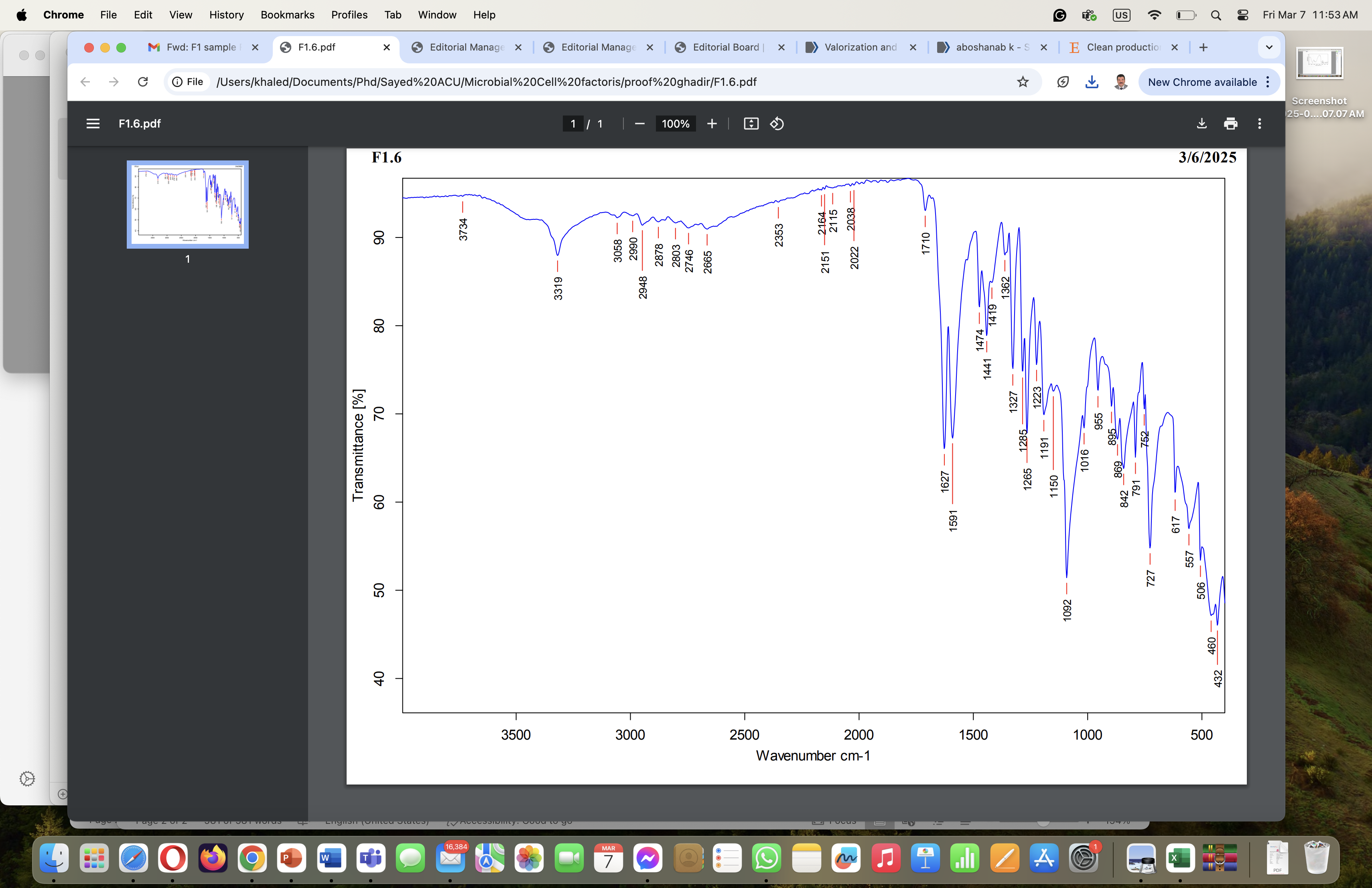


(a)


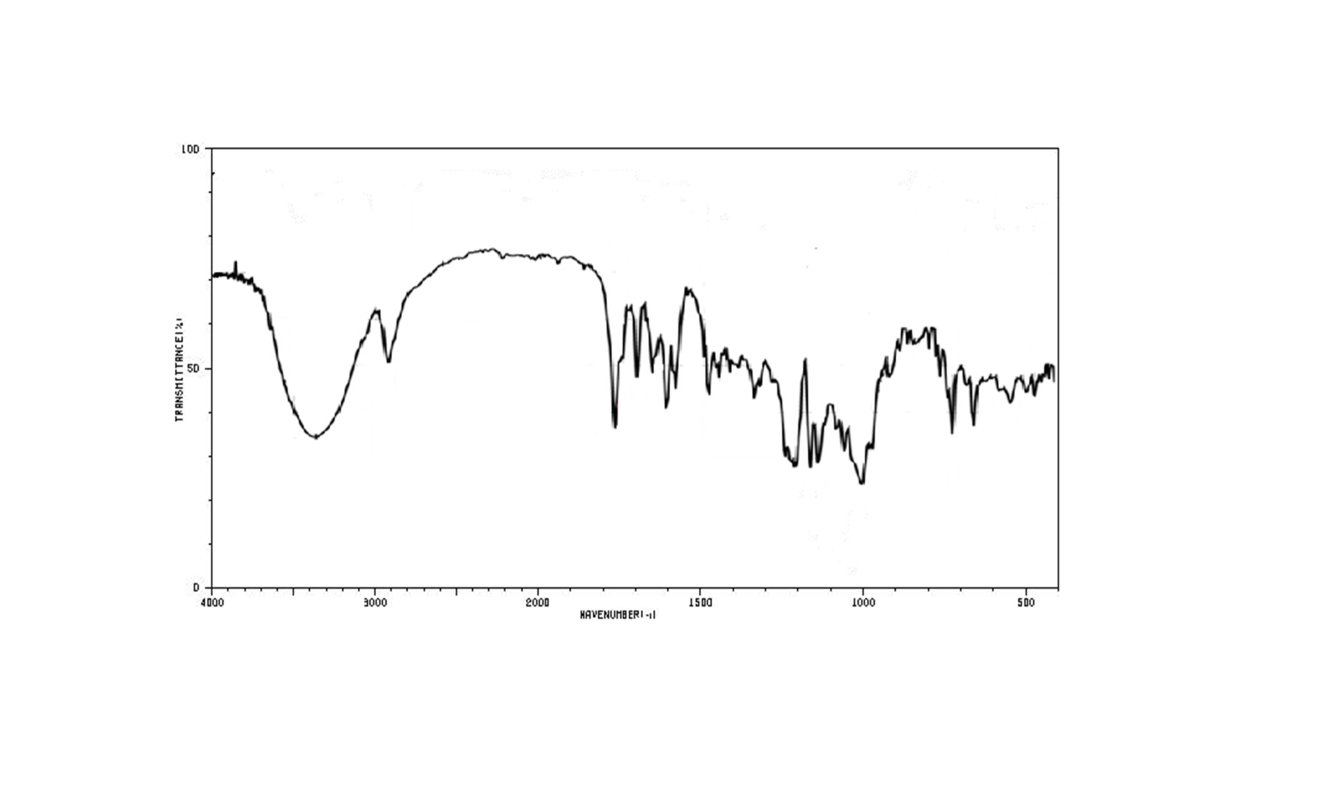


(b)


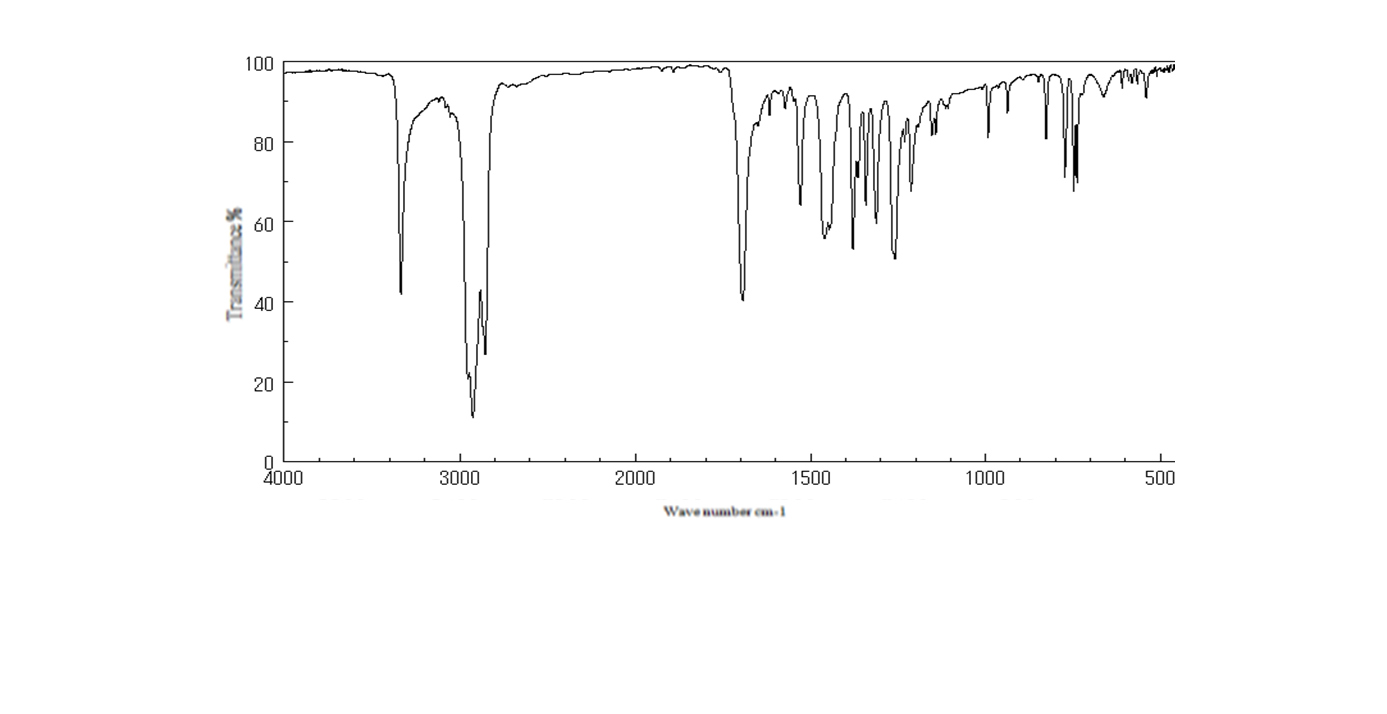


(c)

**Fig. S6** FTIR spectra of (a) MICA-NS complex (b) β-CD and (c) MICA

**Fig. S7** *In vitro* release profile curve of the formulated MICA-NS-HG

**Table S1** Evaluation of different gel formers for the preparation of hydrogel

| **Visual characteristics** | **Sodium alginate** | **CMC** | **Poloxamer 188** | **HPMC E4** | **Carbopol 940** |
| --- | --- | --- | --- | --- | --- |
| Grittiness | Very high | NO | Very high | High | NO |
| Stickiness | Very high | NO | High | High | NO |
| Spreadability | Very low | VERY GOOD | Fairly good | Low | EXCELLENT |
| Formation of clumps | Very high | Low | NO | NO | NO |

**Table S2** Different gel compositions of carbopol 940

| **Component** | **Formulation code** | | | | | |
| --- | --- | --- | --- | --- | --- | --- |
|  | HG-1 | HG-2 | HG-3 | HG-4 | HG-5 | HG-6 |
| Carbopol 940 (w/v %) | 0.2 | 0.5 | 0.8 | 1 | 1.2 | 1.5 |
| Propylene glycol: distilled water ratio (ml) | 30:70 | 30:70 | 30:70 | 30:70 | 30:70 | 30:70 |
| Triethanolamine (ml) | 2 | 2 | 2 | 2 | 2 | 2 |
| MICA -NS | 0.15mg | 0.15mg | 0.15mg | 0.15mg | 0.15mg | 0.15mg |
| Methyl paraben (gram) | 1 | 1 | 1 | 1 | 1 | 1 |
| Distilled water (w/w %) (q.s) | 100 | 100 | 100 | 100 | 100 | 100 |

**Table S3** Evaluation of different carbopol 940 hydrogel formulations

| Visual characteristics | HG1 | HG2 | HG3 | HG4 | HG5 | HG6 |
| --- | --- | --- | --- | --- | --- | --- |
| Grittiness | Yes(+++) | NO | Yes(+) | NO | NO | NO |
| Stickiness | Yes(+++) | NO | Yes(++) | NO | NO | NO |
| Spreadability | Bad | Bad | Fairly good | Good | Fairly good | Good |
| Homogeneity | CLUMPS | YES | YES | Yes | NO | Slightly clumpy |
| Syneresis | Yes(+) | Yes(+++) | NO | No | No | No |
| Color | White | Translucent | White | White | White | White |

**Table S4** Comparison of correlation coefficient (R^2^) of different kinetic models

|  | **Zero order** | **First order** | **Korsmeyer and Peppas** | **Hixson and Crowell model** | **Higuchi ’ s model** |
| --- | --- | --- | --- | --- | --- |
| **R^2^** | 0.8035 | 0.865 | 0.8751 | 0.9182 | 0.9596 |

**Table S5** Stability studies data

| **Mean data** ± **(SD)** | | | **Physical appearance** | **Storage conditions** |
| --- | --- | --- | --- | --- |
| **Drug content** | **Viscosity (cps)** | **pH** |  |  |
| 91.42±0.56% | 1088±2 | 6.42±0.28 | Translucent-smooth homogenous | Initial |
| 88.65±0.21% | 1075±3 | 6.88±0.72 | Translucent -smooth homogenous | Room temperature |
| 86.48±0.52% | 1079±2 | 6.95±0.4 | Translucent -smooth homogenous | 5 ± 3 °C |
